# 7,8-Dihydroxyflavone induces mitochondrial apoptosis and down-regulates the expression of ganglioside GD3 in malignant melanoma cells

**DOI:** 10.1007/s12672-023-00643-0

**Published:** 2023-03-30

**Authors:** Won Seok Ju, Sang Young Seo, Seong-eun Mun, Kyongtae Kim, Jin Ok Yu, Jae-Sung Ryu, Ji-Su Kim, Young-Kug Choo

**Affiliations:** 1 Department of Biological Science, College of Natural Sciences, Wonkwang University, 460, Iksan-daero, Iksan-si, Jeollabuk-do 54538 Republic of Korea; 2 Animal Biotechnology Division, Rural Development Administration, National Institute of Animal Science, 1500 Kongjwipatjwi-ro, Iseo-myeon, Wanju-gun, Jeonbuk 55365 Republic of Korea; 3 Stem Cell Convergence Research Center, Korea Research Institute of Bioscience and Biotechnology (KRIBB), 125, Gwahak-ro, Yuseong-gu, Daejeon, 34141 Republic of Korea; 4 Primate Resources Center (PRC), Korea Research Institute of Bioscience and Biotechnology (KRIBB), 181, Ipsin-gil, Jeongeup-si, Jeollabuk-do 56216 Republic of Korea; 5 Institute for Glycoscience, Wonkwang University, 460, Iksan-daero, Iksan-si, Jeollabuk-do 54538 Republic of Korea

**Keywords:** 7,8-dihydroxyflavone, Anti-cancer effects, Mitochondrial apoptosis, Ganglioside GD3, Malignant melanoma

## Abstract

Malignant melanoma is a skin cancer with poor prognosis and high resistance to conventional treatment. 7,8-dihydroxyflavone (7,8-DHF) has shown anti-carcinogenic, anti-inflammatory, anti-oxidant, and pharmacological effects in several types of cancer. However, the relationship between ganglioside expression and the anti-cancer effects of 7,8-DHF in melanoma is not fully understood. In the present study, 7,8-DHF exhibits specific anti-proliferation, anti-migration, and G2/M phase cell-cycle arrest effects on both melanoma cancer cell lines, and induces mitochondrial dysfunction and apoptosis, making it a potent candidate for anti-melanoma treatment. Furthermore, we confirmed that 7,8-DHF significantly reduces the expression levels of ganglioside GD3 and its synthase, which are known to be closely involved in carcinogenesis. Taken together, our findings suggest that 7,8-DHF may be a potent anti-cancer drug candidate for the treatment of malignant melanoma.

**Supplementary Information:**

The online version contains supplementary material available at 10.1007/s12672-023-00643-0.

## Introduction

Malignant melanoma worldwide is a highly perilous form of representative skin cancers including non-melanomas (basal cell carcinoma and squamous cell carcinoma), which leads to poor prognosis with limited survival rates and unsuccessful therapy approaches due to their substantial side effects and significant toxicities for patients [1–4]. Despite the advancements in cancer therapy with the addition of immunotherapy and targeted drugs, the response rate remains unpredictable and dependent on the molecular and genetic characteristics of the tumor [5–7]. For an example, despite revolutionizing cancer treatment, ani-cancer immunotherapy faces major challenges including limited efficacy, high costs, significant side effects, and lack of accurate diagnostic bio-markers [8]. Therapeutic molecular targets specific to a patient’s melanoma are still needed to be developed.

7,8-Dihydroxyflavone (7,8-DHF) is a natural polyphenolic compound found in several plants with therapeutic efficacy in various central nervous system disorders [9–12]. It also possesses anti-proliferative and anti-cancer effects in different type of cancers, and acts as a powerful anti-oxidative agent [13]. Additionally, 7,8-DHF has bioactivities such as protecting against excitotoxicity, interacting with the main signaling receptor of brain-derived neurotrophic factor, and exhibiting anti-inflammatory, anti-mutagenic effect [11, 12, 14–16]. Moreover, several researches revealed that 7,8-DHF induce various mechanisms such apoptosis, G1 phase cell-cycle arrest, regulation of specific protein 1, mitochondrial dysfunction, MAPKs signaling pathway and activate TrkB-Akt signaling in different type of cancers [13, 17–21]. The development of anti-cancer agents from natural compounds, such as quercetin, is an important topic. However, the mechanism underlying the anti-cancer effects of 7,8-DHF is not fully understood.

Gangliosides are normally abundant in the central and peripheral nervous systems [22]. It plays an important role in cellular interactions such as cell growth, proliferation, cellular recognition, cell-to-cell communication, differentiation, and motility [23–25]. Several studies revealed that the expression of specific gangliosides, especially ganglioside GD3, was increased in several types of cancer including melanoma, colorectal cancer, breast cancer, and hepatocellular carcinomas [23, 24, 26, 27]. In addition, osteosarcoma cells and tissues from patients showed over-expression of ganglioside GD3 [28, 29]. Furthermore, the expression of ganglioside GD3 and its synthase is involved in cancer development and progression, and in mitochondrial apoptosome activation as an apoptotic effector [30–34]. However, researches on the relationship between the expression of ganglioside and pharmacological roles of 7,8-DHF in melanoma is still insufficient.

To investigate whether 7,8-DHF regulates anti-melanoma effects (cancer development and progression) and changes the expression level of ganglioside profiles, especially ganglioside GD3, which was analyzed using high-performance thin-layer chromatography. Our findings suggest 7,8-DHF has 1) potential chemotherapeutic efficacy against melanoma cells, and 2) involves in the synthesis of ganglioside in melanoma.

## Materials and methods

### Reagents and antibodies

7,8-dihydroxyflavone (7,8-DHF) was purchased from Tokyo Chemical Industry (TCI; Tokyo, Japan) and was dissolved in dimethyl sulfoxide. Unless otherwise stated, all other reagents were purchased from Sigma-Aldrich (St. Louis, MO, USA). The following antibodies from Sigma-Aldrich (St. Louis, MO, USA) were used: anti-p53 (C-11), anti-p21 (F-5), anti-cyclin B (GNS1), anti-CDK1 (AN21.2), anti-Mcl-1 (22), anti-survivin (D-8), anti-Bax (P-19), anti-caspase 3 (L-18), anti-caspase 9 (96.1.23), anti-cleaved-caspase 3 (h176), anti-cytochrome c (A-8), anti-GM3 synthase (T-19), anti-GD3 synthase (B-11), anti-Bcl-xL (H-5), and anti-β-actin (C-4) (Santa Cruz Biotechnology, Inc., Santa Cruz, CA, USA). The other antibodies (anti-Bid, anti-PARP, anti-MTCO1, and anti-tubulin β) were purchased from Cell Signaling Technology (Danvers, MA, USA) and Novus Biologicals, LLC (Centennial, CO, USA).

### Cell culture

The SK-MEL-2 and G-361 human malignant melanoma cell lines were obtained from the Korean Cell Line Bank (Seoul, Korea). SK-MEL-2 and G-361 cells were maintained in Dulbecco’s modified Eagle’s medium (DMEM; Welgene, Gyeongsan, Gyeongbuk, Korea) supplemented with 10% fetal bovine serum (FBS; Gibco, Gaithersburg, MD, USA) and 100 U/mL penicillin–streptomycin (PS; Gibco, Gaithersburg, MD, USA) at 37 ℃ under 5% CO_2_ in a humidified incubator chamber until the experiments were performed. HaCaT cells, a non-tumoral human epidermal cell line, were kindly provided by Prof. Jong Kun Park (Wonkwang University, Iksan, Jeonbuk, Korea). HaCaT cells were maintained in RPMI-1640 medium (Welgene, Gyeongsan, Gyeongbuk, Korea) supplemented with 10% FBS and 100 U/mL of PS at 37 ℃ and 5% CO_2_ in a humidified incubator chamber until the next use.

### MTS cell viability assay

The effect of 7,8-DHF on the viability of malignant melanoma cells was determined using the 3-(4,5-dimethyl-thiazol-2-yl)-5-(3-carboxymethoxyphenyl)-2-(4-sulfophenyl)-2H-tetrazolium) (MTS) assay kit (Promega, Madison, WI, USA), according to the manufacturer’s instructions. Briefly, SK-MEL-2 (1.0 × 10^3^ cells/well), G-361 (1.0 × 10^3^ cells/well), and HaCaT cells (1.0 × 10^3^ cells/well) were seeded in 96-well plates (Thermo Fisher Scientific lnc., Waltham, MA, USA) for 24 h (hr) and treated with 7,8-DHF (50, 100, 150, 200, 250, and 300 µM) for 24 or 48 h. MTS solution was then added for three hours at 37 ℃ in a 5% CO_2_ incubator. The absorbance was finally measured at 490 nm and recorded using a BioTek Synergy HTX-Multi Microplate Multimode Reader (Agilent, CA, USA).

### Nuclear staining with DAPI

The levels of nuclear condensation and fragmentation in malignant melanoma cells were confirmed by using nucleic acid staining and DAPI staining. Melanoma cell line (SK-MEL-2 and G-361) and HaCaT cells treated with 7,8-DHF at different concentration (0, 100, 200, and 300 µM for 24 h) were harvested by trypsinization, and fixed in 4% paraformaldehyde (Sigma, St. Louis, MO, USA) in Dulbecco’s modified phosphate-buffered saline (DPBS; Welgene, Gyeongsan, Gyeongbuk, Republic of Korea) at room temperature for 30 min. The cells were spread on slides, stained with DAPI solution (2 µg/mL), and analyzed under a fluorescence microscope (Carl Zeiss, Ulm, Germany).

### Cell cycle analysis

For facile and quantitative measurements of the percentage of cells in a variety of cell cycles, both melanoma SK-MEL-2 (1.0 × 10^5^ cells/well) and G-361 cells (1.0 × 10^5^ cells/well) were seeded into 6-well plates (Thermo Fisher Scientific lnc., Waltham, MA, USA) and maintained for 24 h. After exposure to 7,8-DHF at different concentrations (100, 200, and 300 µM) for 24 h, the cells were detached by trypsinization and washed twice with DPBS. Detached samples were fixed in 70% ethanol at – 20 ℃ until staining. Fixed samples were then washed with DPBS, collected by centrifugation at 300 × g for 5 min, and mixed with 150 µL Muse^™^ Cell Cycle Reagent (Luminex Corp., TX, USA). After suspension, the mixed samples were incubated at room temperature for 30 min and protected from light. Cells were analyzed by flow cytometry using a Muse^™^ Cell Analyzer (Merck Millipore, MA, USA).

### Annexin v & dead cell assay

For the quantitative analysis of live cells, early and late apoptosis, and cell death in melanoma cells, both SK-MEL-2 (3.1 × 10^5^ cells/well) and G-361 (4.3 × 10^5^ cells/well) cells were seeded on 6-well plates (Thermo Fisher Scientific lnc., Waltham, MA, USA) and cultured for 24 h. After treatment with different concentrations (100, 150, 200, 250, and 300 µM) of 7,8-DHF for 24 h, the cells were stained with Muse^™^ Annexin V & Dead Cell kit (Luminex Corp., TX, USA) for 20 min at room temperature in the dark. Stained samples were analyzed by using a Muse^™^ Cell Analyzer (Merck Millipore, MA, USA).

### Reverse transcription-polymerase chain reaction (RT-PCR)

Total RNA was extracted from melanoma cell lines (SK-MEL-2 and G-361) treated with 7,8-DHF at different concentrations (0 and 200 µM for 24 h) by using TRIzol^™^ Reagent (ThermoFisher Scientific lnc., Waltham, MA, USA), and 1.5 µg of RNA was used to synthesize cDNA using the AccuPower® RT PreMix kit (Bioneer Corp., Daejeon, Korea). The cDNA for RT-PCR was obtained by the amplification using specific primers such as β-actin, ST3GAL5, and ST8SIA1. For quantitative PCR, 2 µL of cDNA, 10 pmol of the forward primer, 10 pmol of the reverse primer, and 5 µL of 20X premix buffer were added with distilled water to a final volume of 20 µL. The mixture was under the following conditions: 94 °C for 5 min, followed by 35 cycles of 95 °C for 30 s, 62 °C for 30 s, and 72 °C for 30 s, with a final extension at 72 °C for 10 min. Primer information and the specific PCR conditions used in this study are provided in Additional file 1: Table S1.

### Mitochondrial membrane potential (MMP) assay

MMPs were measured and stained using the JC-1 MMP Assay kit (Abcam Inc., Cambridge CB2, UK) according to the manufacturer’s instructions. Briefly, melanoma SK-MEL-2 (1.0 × 10^5^ cells/well) and G-361 cells (1.0 × 10^5^ cells/well) were first cultured and then treated with 7,8-DHF at different concentrations (100–200 µM) for 24 h. Cells were washed twice with DPBS, added 5 µM JC-1 in 1 × dilution buffer, and then incubated at 37 ℃ for 15 min in the dark. The samples were washed twice with DPBS. MMPs of the mitochondria in the cancer cells were observed using a fluorescence microscope (Carl Zeiss, Ulm, Germany). The aggregated form of JC-1 yielded a red to orange colored emission (590 ± 17.5 nm), whereas the monomeric form yielded a green colored emission of 530 ± 15 nm.

### Isolation of mitochondria and protein fractionation

Mitochondrial and cytosolic fractions were isolated using a Mitochondria and Cytosol Fractionation Kit (Abcam Inc., Cambridge CB2, UK), according to the manufacturer’s instructions [46]. After treatment with 7,8-DHF for up to 24 h, both melanoma SK-MEL-2 and G-361 cells were harvested and then mixed with 1 mL of 1 × cytosol extraction buffer mix containing DTT and protease inhibitors. After incubation on ice for 10 min, the cell samples were homogenized in an ice-cold Dounce glass tissue grinder (DWK Life Science, St. Modwen Park, Lincoln LN6 9BJ, UK) for 40–60 passes. The samples were then centrifuged at 700 × g at 4 ℃ for 10 min, the supernatant was collected, and the pellet was discarded. The supernatants were centrifuged at 10,000 × g at 4 ℃ for 30 min to separate the mitochondrial (pellets) and cytosolic (supernatants) fractions. Finally, mitochondrial pellets were suspended in 150 µL of mitochondrial extraction buffer mix containing DTT and protease inhibitors. All isolated samples were stored at − 80 ℃ until use.

### Western blot analysis

SK-MEL-2 and G-361 cells were treated with various concentrations of 7,8-DHF for different incubation times (100, 200, and 300 µM, for 12 and 24 h) and washed twice with DPBS. Cells were homogenized well with a protein extraction solution (RIPA) (ELPIS Biotech Inc., Daejeon, Korea). The extracted proteins were quantified using the Pierce^®^ BCA Protein Assay kit (Thermo Fisher Scientific lnc., Waltham, MA, USA). Equal amounts of protein samples were separated by 8%, 10%, and 15% sodium dodecyl sulfate–polyacrylamide gel electrophoresis (SDS-PAGE) along with protein markers and then transferred to polyvinylidene difluoride (PVDF) blotting membranes. The membranes were blocked at room temperature for one hour with 5% non-fat dried milk in 1 × TBS (Biosesang, Seongnam, Gyeonggi, Korea) containing 0.1% Tween-20 (Bio-Rad Inc., CA, USA) and then incubated overnight at 4 ℃ with specific primary antibodies. After incubation with horseradish peroxidase-conjugated secondary antibodies at room temperature for two hours, immunoreactive bands were visualized using a SuperSignal West Pico PLUS chemiluminescent substrate (ThermoFisher Scientific lnc., Waltham, MA, USA).

### Wound healing assay

Confluent SK-MEL-2 (5.0 × 10^5^ cells/well) and G-361 cells (5.0 × 10^5^ cells/well), grown in 60 mm culture dishes (Thermo Fisher Scientific lnc., Waltham, MA, USA), were sharply scratched using a sterile tip, and each well was washed twice with DPBS to remove cell debris. After scratching, the cells were treated with 200 µM 7,8-DHF and then cultured for 24, 48, and 72 h to observe the perimeter of the area with a central cell-free zone under an inverted phase-contrast microscope (Carl Zeiss, Ulm, Germany).

### Ganglioside extraction and purification

Gangliosides from melanoma cell lines (SK-MEL-2 and G-361) and HaCaT cells treated with 7,8-DHF at different concentration (0 and 200 µM for 24 h) was prepared as previously described [35]. Briefly, total lipids were extracted using chloroform/methanol (1:1, v/v). Next, neutral lipids were filtered off with 10 mL chloroform/methanol/H_2_O (15:30:4, v/v/v) by applying a DEAE Sephadex A25 column (Sigma, St. Louis, MO, USA). Acidic lipids were then extracted with 5 mL chloroform/methanol/0.8 M sodium acetate (15:30:4, v/v/v). The eluted samples were dried with N_2_ gas at 30 ℃, dissolved in chloroform/methanol (1:1, v/v), and incubated for neutralization with 12N NH_4_OH overnight at room temperature. After the neutralized samples were dried with N_2_ gas, they were dissolved in distilled water and the salt was removed using a Sep-Pak C18 cartridge (Merck Millipore, MA, USA) to obtain gangliosides. The eluted gangliosides were dried under N_2_ gas at 30 °C. Finally, the dried samples were stored at − 80 ℃ until further experiments.

### performance thin-layer chromatography (HPTLC)

HPTLC analysis by using eluted ganglioside was performed as previously described [35]. 10 µg/µL of purified gangliosides in chloroform/methanol (1:1, v/v) were spotted using a capillary tube on 10 × 10 cm TLC plates (Merck Millipore, MA, USA) and then developed with 100 mL chloroform/methanol/0.25% CaCl_2_·H_2_O (50:40:10, v/v/v). The developed gangliosides were stained with resorcinol solution [HCl (Samchun, Seoul, Korea), 0.1 M CuSO_4_·5H_2_O (Sigma-Aldrich, St. Louis, MO, USA), Resorcinol (Sigma-Aldrich, St. Louis, MO, USA), distilled water], and the stained samples on the HPTLC plate were dried at 105 ℃ in a dry-oven for at least three hours. Monosialoganglioside (Matreya LLC, State College, PA, USA) and disialoganglioside (Matreya LLC, State College, PA, USA) mixtures were used as standard markers. The quantitation of ganglioside expression of HPTLC was used densitometry program (Beta 4.0.3 from Scion Image, Frederick, MD, USA).

### Data analysis

For statistical analysis, all data were analyzed for statistical significance with One-way ANOVA, post hoc Tukey’s multiple range test by using the Statistical Package for the Social Science statistics software (IBM SPSS Corp., Armonk, NY, USA) (ver. 25). Other data are represented as mean ± standard deviation (SD). A *p* value (*p* < 0.05) in this study was considered statistically significant.

## Results

### 7, 8-dihydroxyflavone (7,8-DHF) reduced viabilities and induced cellular morphology in melanoma cells

**Fig. 1:**
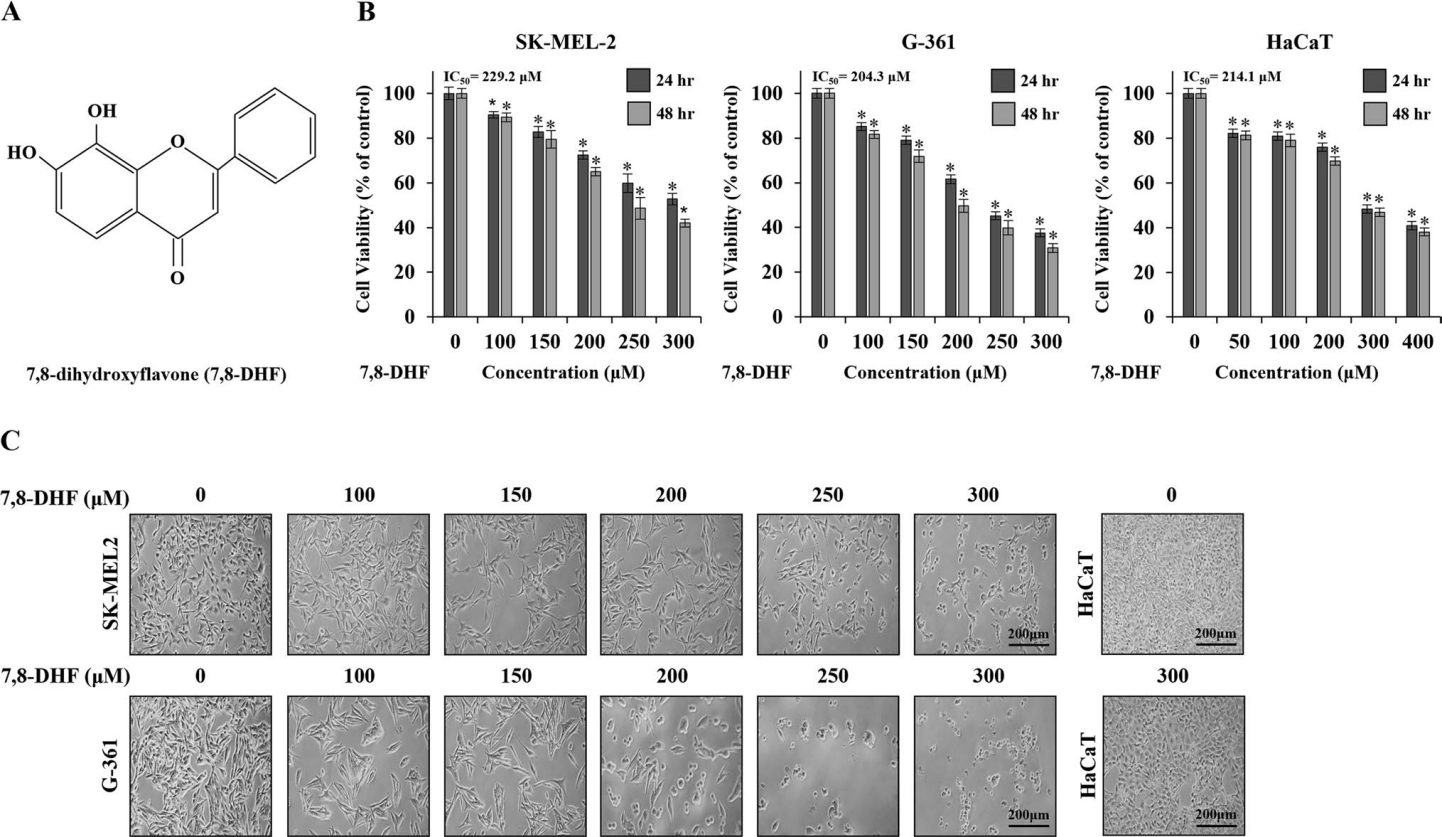
The effects of 7,8-dihydroxyflavone (7,8-DHF) on viability in malignant melanoma cells and HaCaT cells. **A** Chemical structure of 7,8-DHF. **B** The inhibitory effect of 7,8-DHF on the proliferation of melanoma cells. SK-MEL-2 (1.0 × 10^3^ cells/well), G-361 (1.0 × 10^3^ cells/well), and non-tumoral HaCaT cells (1.0 × 10^3^ cells/well) were treated with different concentrations of 7,8-DHF for 24 and 48 h (hr). The viability was measured using an MTS assay kit. **C** Morphological changes in 7,8-DHF-treated (24 h) SK-MEL-2, G-361, and HaCaT cells and the corresponding untreated controls. Data represented the mean ± standard deviation (SD) of three independent experiments. (^*^p < 0.05)

To determine whether 7,8-DHF affects the viability of melanoma cells, SK-MEL-2, G-361, and non-tumoral epidermal HaCaT cells were treated with various concentrations of 7,8-DHF at two time points [24 and 48 h (hr)], and an MTS assay was performed. The chemical structure of 7,8-DHF used in this study is shown in Fig. 1A. The half maximal inhibitory concentrations (IC_50_) reducing cell viability by 50% in SK-MEL-2 and G-361 cells were 229.2 µM and 204.3 µM, respectively. In contrast, the IC_50_ for HaCaT cells as the control cell line was 345.7 µM (Fig. 1B). The inhibition of viability by 7,8-DHF was significant in both melanoma cell lines when the concentration ranged from 200 to 250 µM. In HaCaT cells, no significant change was observed while HaCaT cells were exposed up to 300 µM. To analyze morphological changes, SK-MEL-2 and G-361 cells were treated with 7,8-DHF (100–300 µM, for 24 h). The number of cells was markedly decreased, and the cell size also decreased and became more rounded (Fig. 1C). In contrast, HaCaT cells showed no distinctive morphological changes after treatment with 300 µM 7,8-DHF.

### 7,8-DHF induced apoptosis in melanoma cells

**Fig. 2:**
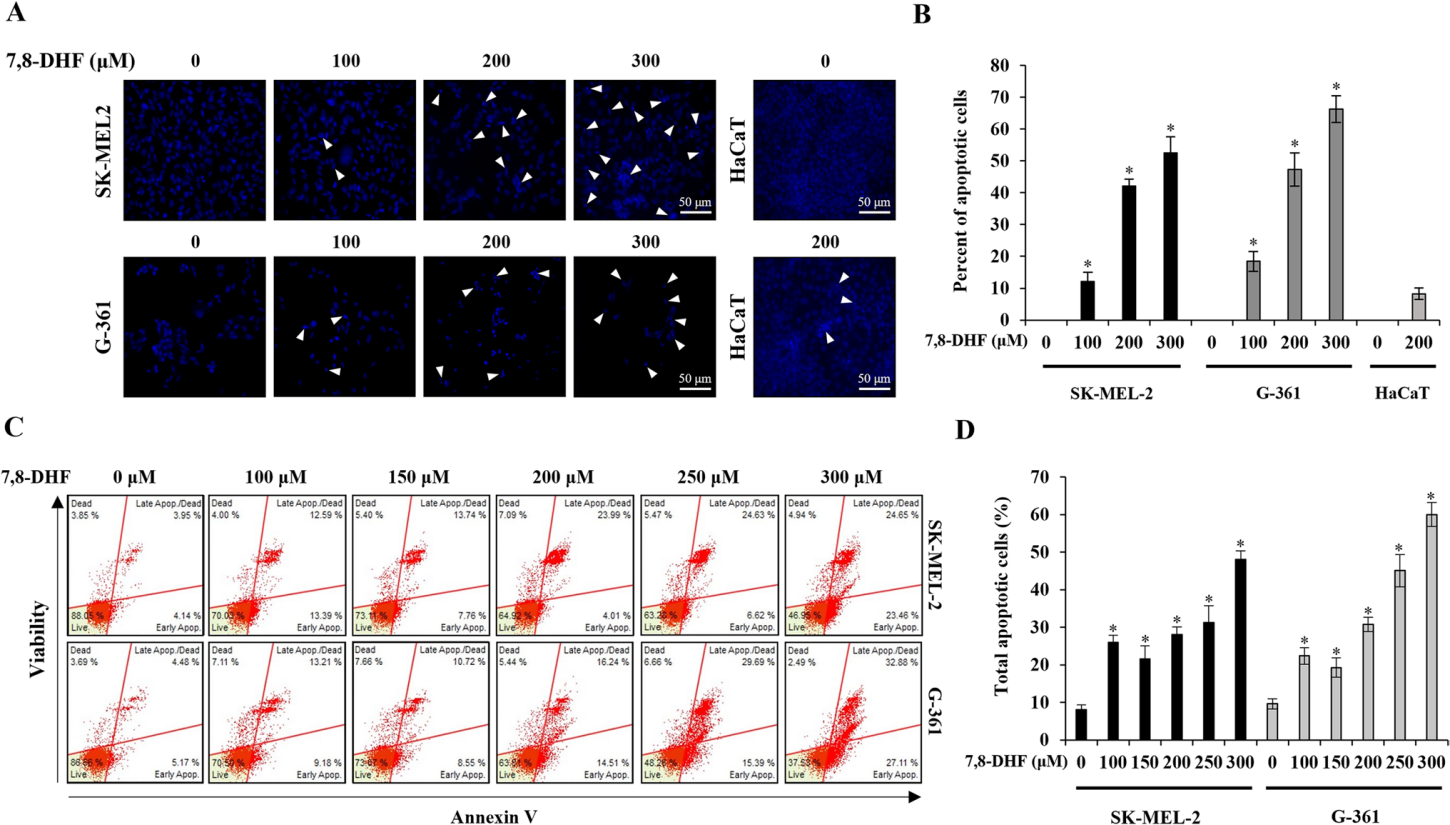
Apoptotic effects induced by 7,8-DHF in malignant melanoma cells. SK-MEL-2, G-361, and HaCaT cells were maintained without 7,8-DHF (control group) or with different concentrations of 7,8-DHF (24 h). **A** Fluorescence microscopy images (original magnification: × 200) of nuclear staining with DAPI. White arrows represent DNA fragmentation and nuclear condensation. **B** Quantification of DNA fragmentation and condensation in melanoma cells. **C** Quantitative detection of Annexin-V/7-ADD positive cells was performed using a Muse^™^ Cell analyzer. SK-MEL-2 (3.1 × 10^5^ cells/well) and G-361 (4.3 × 10^5^ cells/well) cells were treated with 7,8-DHF (100, 150, 200, 250, and 300 µM, 24 h). Cells stained with Annexin-V only were defined as early apoptotic, whereas Annexin-V and 7-ADD double-stained cells were defined as late apoptotic. **D** The percentage of the total apoptotic cell population was quantified. Data were presented as mean percentage levels ± SD (*n* = 3, ^*^p < 0.05)

The effects of 7,8-DHF on cellular apoptosis in SK-MEL-2, G-361, and HaCaT cells were determined by nuclear morphology using DAPI staining and Annexin-V/7-ADD staining analysis, and these results were verified by the images of the morphological changes. It was confirmed that 7,8-DHF (100, 200, and 300 µM for 24 h) induced nuclear condensation and perinuclear apoptotic bodies in melanoma cells in a concentration-dependent manner. Morphological and nuclear changes were also induced in HaCaT cells by 7,8-DHF (200 µM), although to a lower degree (Fig. 2A). The percentage of cells with nuclear fragmentation in 7,8-DHF-treated cells vs. non-treated cells is shown in Fig. 2B. Additionally, the apoptotic effect of 7,8-DHF on SK-MEL-2 and G-361 melanoma cells was evaluated after 24 h using the Annexin-V/7-ADD assay (Fig. 2C). For the upper panel in Fig. 2C, the average percentage of early apoptotic cells in the SK-MEL-2 group was 4.14, 13.39, 7.76, 4.01, 6.62, and 23.46% after treatment with 0, 100, 150, 200, 250, and 300 µM 7,8-DHF, respectively, and the average ratio of dead cells and late apoptotic cells was 3.95, 12.59, 13.74, 23.99, 24.63, and 24.65%, respectively. The proportion of live cells in the control group was 88.05%, which decreased to 70.03, 73.11, 64.92, 63.28, and 46.95% as the concentration of 7,8-DHF increased. Similarly, the lower panel in Fig. 2C shows the proportions in the G361 group. The average percentage of cells in the early apoptosis phase was 5.17, 9.18, 8.55, 14.51, 15.39, and 27.11% after treatment with 0, 100, 150, 200, 250, and 300 µM 7,8-DHF, respectively, and the average percentage of dead cells and late apoptotic cells was 4.48, 13.21, 10.72, 16.24, 29.69, and 32.88%, respectively. The proportion of live cells in the control group was 86.86%, which decreased to 70.5, 73.07, 63.81, 48.26, and 37.53% as the concentration of 7,8-DHF increased. The percentage of the total apoptotic cell population in 7,8-DHF-treated cells vs. non-treated cells is shown in Fig. 2D.

### 7,8-DHF induced cell cycle arrest and regulated apoptosis-related proteins in melanoma cells

**Fig. 3:**
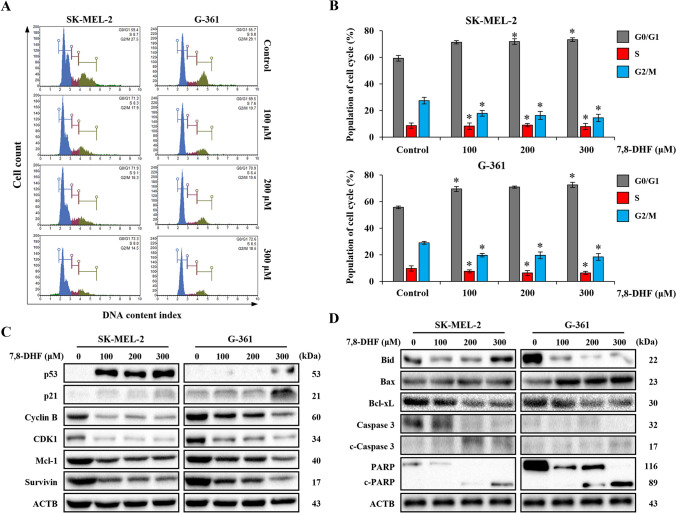
Anti-proliferative and apoptotic effects induced by 7,8-DHF in malignant melanoma cells. **A** SK-MEL-2 (1.0 × 10^5^ cells/well) and G-361 cells (1.0 × 10^5^ cells/well) were incubated without 7,8-DHF (control group) or with 7,8-DHF (100, 200, and 300 µM for 24 h). Cells were then washed with DPBS, fixed in 70% ethanol overnight, stained with Muse^™^ Cell-Cycle reagent, and finally analyzed for DNA content using a Muse^™^ Cell analyzer. **B** The results of cell cycle population in SK-MEL-2 and G-361 cells were quantified. Data represented the mean ± SD (*n* = 3, ^*^p < 0.05). **C** SK-MEL-2 and G-361 cells were treated with different concentrations of 7,8-DHF (100, 200, and 300 µM for 24 h). Whole cells extracts were prepared, separated on SDS-PAGE, and transferred onto blotting membranes. Western blot analysis was performed using p53, p21, Cyclin B, CDK1, MCL-1, and Survivin antibodies. **D** SK-MEL-2 and G-361 cells were treated with 7,8-DHF (100, 200, and 300 µM for 24 h). The lysate samples were subjected to western blot analysis with BID, BAX, BCL-_X_L, Caspase 3 (CASP3), cleaved-CASP3, poly (ADP-ribose) polymerase (PARP), and cleaved-PARP antibodies. β-actin was used as a loading control. The results shown in **C**, **D** were representative of at least three independent experiments

To investigate the inhibitory effect of 7,8-DHF on cell cycle arrest in SK-MEL-2 and G-361 cells, both cells were treated with 7,8-DHF for 24 h, and the cell cycle distribution was determined by FACS analysis (Fig. 3A, B). In SK-MEL-2 cells, the levels of G2/M cells were 27.5%, 17.9%, 16.3%, and 14.5% when treated with 0, 100, 200, and 300 µM 7,8-DHF, respectively (left panel in Fig. 3A). In G-361 cells, the levels of G2/M cells were 29.1%, 19.7%, 19.6%, and 18.5% when treated with 0, 100, 200, and 300 µM 7,8-DHF, respectively (right panel in Fig. 3A). Thus, 7,8-DHF dose-dependently decreased the levels of G2/M cells in SK-MEL-2 (1.9-fold) and G-361 (1.57-fold), respectively (Fig. 3B).

To verify this change of cell-cycle, the levels of G2/M phase-related proteins (p53, p21, cyclin B, CDK1) were analyzed. As shown in Fig. 3C, The expression levels of p53 and p21 were increased, whereas the expression levels of CDK1 and cyclin B were decreased compared to those in the control. Also, the expression levels of Mcl-1 and Survivin were slightly decreased in dose-dependent manner (Fig. 3C).

To further investigate the relationship between the changes of cell-cycle and cell apoptosis, the apoptosis-related proteins, including Bid, Caspase 3 (CASP3), and poly (ADP-ribose) polymerase (PARP), were analyzed; they were truncated dramatically. Specifically, the expression levels of Bax, cleaved-CASP3, and cleaved-PARP were highly increased, whereas the expression of the anti-apoptotic protein BCL-xL was decreased as shown in Fig. 3D.

### 7,8-DHF changed the Mitochondrial membrane potential (MMP) and induced the release of cytochrome c from the intermembrane space in melanoma cells

**Fig. 4:**
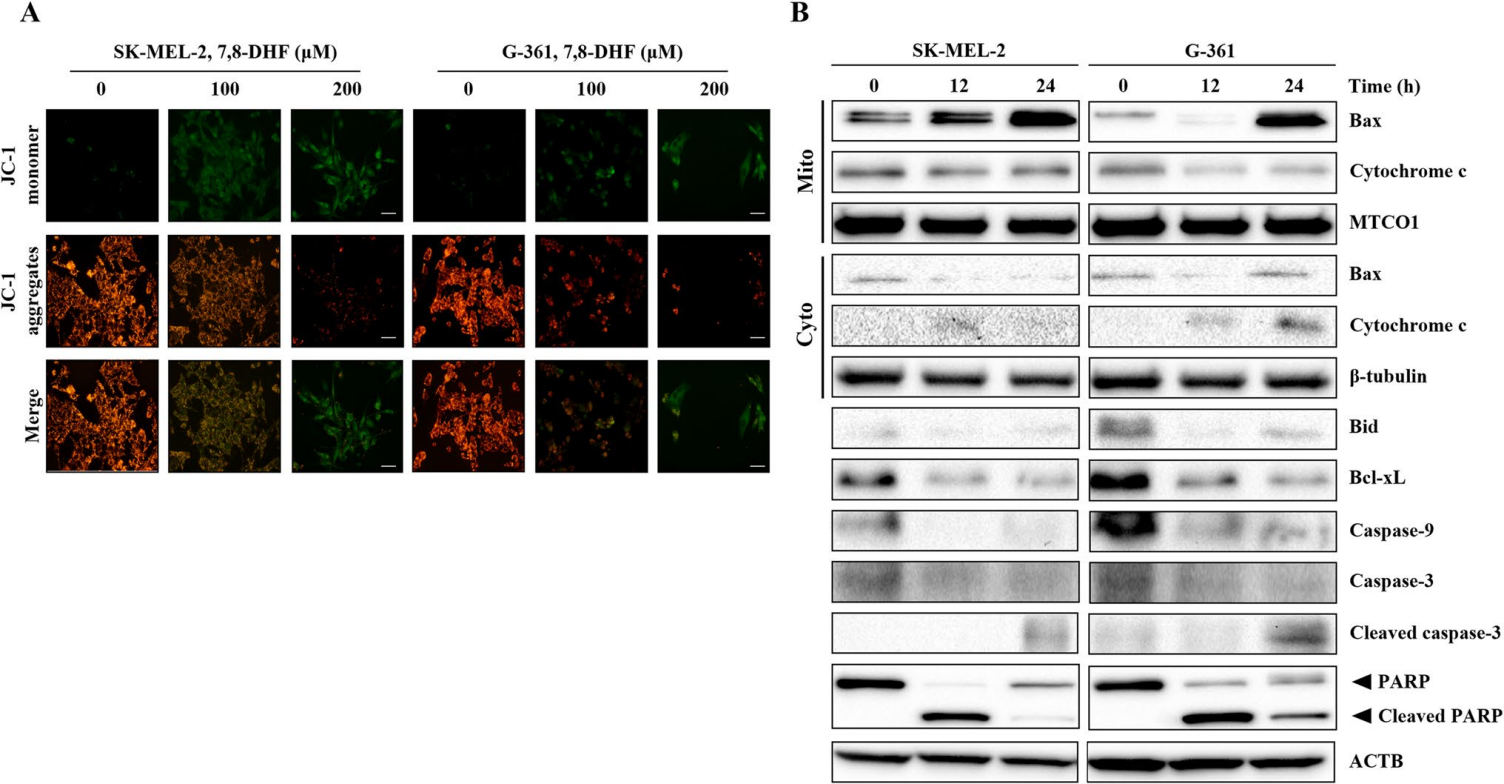
Changes in the mitochondrial membrane potential of malignant SK-MEL-2 and G-361 melanoma cells. **A** Mitochondrial membrane depolarization by using JC-1 staining was determined through the immunofluorescence microscopy. Melanoma SK-MEL-2 (1.0 × 10^5^ cells/well) and G-361 cells (1.0 × 10^5^ cells/well) were treated with 7,8-DHF (100 and 200 µM) for 24 h and mitochondrial membrane depolarization was found to increase in a dose-dependent manner. **B** The mitochondrial apoptotic process in SK-MEL-2 and G-361 cells was determined by western blot analysis of mitochondrial and cytosolic protein levels. The mitochondrial fraction was confirmed using the mitochondrial marker MTCO1, and the cytosolic fraction was confirmed using β-tubulin. 7,8-DHF induced Cytochrome c release and Bax expression. 7,8-DHF decreased BID, BCL-_X_L, Caspase 9, CASP3, and PARP expression time-dependently. Cyto, cytosolic fraction; Mito, mitochondrial fraction

To investigate whether 7,8-DHF-mediated apoptosis in SK-MEL-2 and G-361 cells through the mitochondrial pathway, we measured the MMP using JC-1 staining, and examined the level of Bcl-2 family proteins and cytochrome c. As shown in Fig. 4A, JC-1 monomer (green) was dose-dependently increased, indicating depolarization of the MMP in melanoma cells.

Next, we investigated the expression levels of fractionations into mitochondrial and cytosolic proteins related to the mitochondrial apoptotic pathway in both cells. As shown in Fig. 4 B, 7,8-DHF induced mitochondrial cytochrome c release into the cytosol in SK-MEL-2 and G-361 cells in a time-dependent manner, which was followed by an increase in mitochondrial Bax. In addition, the expression levels of cleaved-CASP3 and cleaved-PARP were dramatically increased, whereas the expression levels of pro-apoptotic proteins including Bid and Caspase-9 and the anti-apoptotic protein Bcl-xL were highly decreased.

### 7,8-DHF inhibited melanoma cell population and migratory capacity

**Fig. 5:**
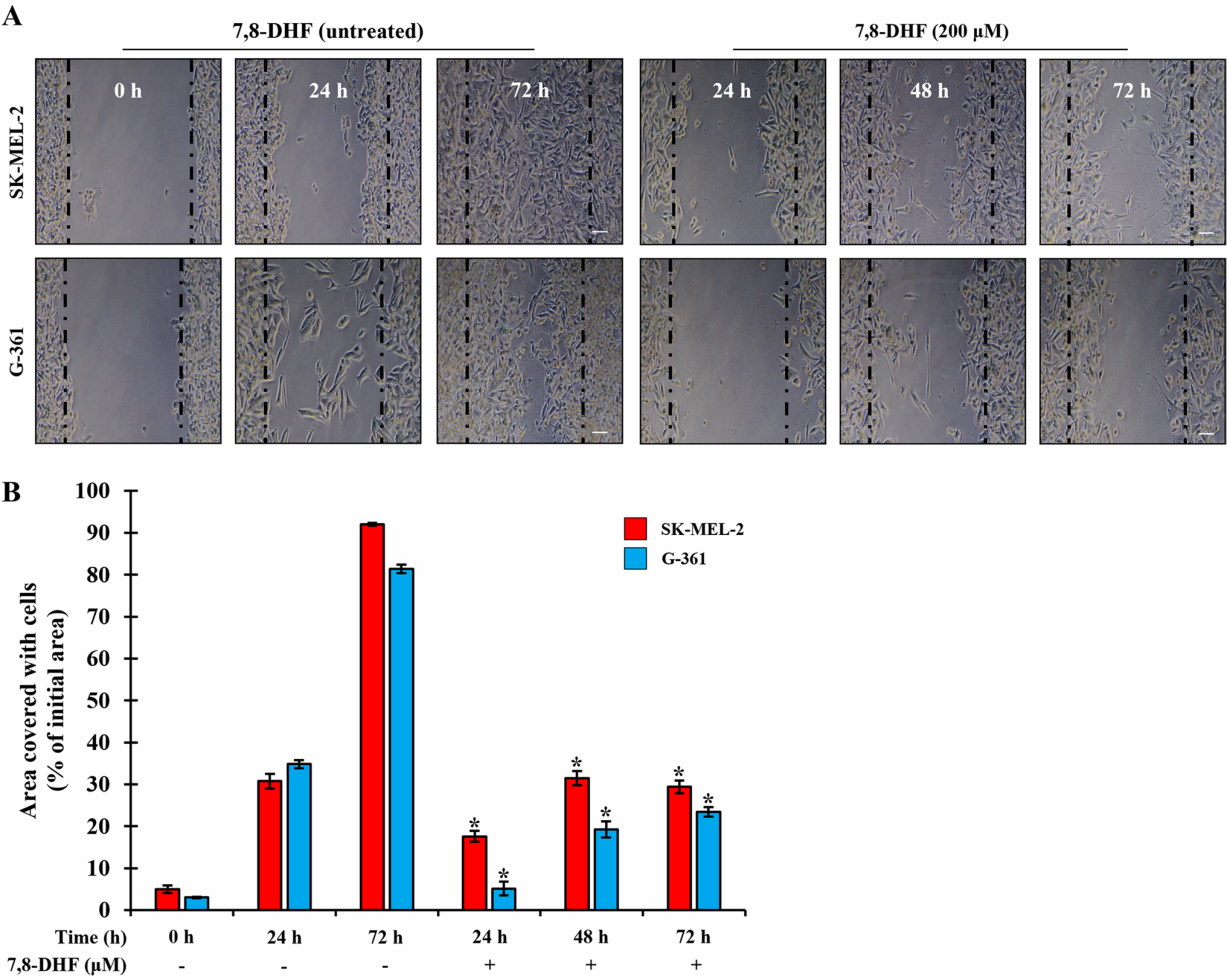
7,8-DHF significantly inhibited the metastatic ability of SK-MEL-2 and G-361 cells. **A** Effects of 7,8-DHF on the migration of melanoma cell lines. SK-MEL-2 (5.0 × 10^5^ cells/well) and G-371 cells (5.0 × 10^5^ cells/well) were treated with 7,8-DHF (200 µM). Melanoma cells were scratched using sterile pipette tips and then incubated with or without (control group) 7,8-DHF. Samples were observed under a light microscope at 24, 48, and 72 h. **B** The area covered with cells was quantified. Red square and blue square represent SK-MEL-2 and G-361 cells, respectively. All data were presented as the mean ± SD (*n* = 3, ^*^p < 0.05)

In most cancer cells, the ability to invade surrounding tissues and other parts of the body is the initial step in tumor metastasis and migration. However, the inhibitory effects of 7,8-DHF on SK-MEL-2 and G-361 melanoma cells remain unclear. Therefore, we examined the effect of 7,8-DHF (200 µM) on the migratory capacity of melanoma cells using a wound healing assay. As shown in Fig. 5A, treatment with 7,8-DHF reduced the migration capacity of SK-MEL-2 and G-361 cells. 7,8-DHF-treated SK-MEL-2 cells covered 17.6%, 31.47%, and 29.38% of the scratched area at 24, 48, and 72 h after scratching, respectively (upper panel in Fig. 5A , B). Similarly, 7,8-DHF-treated G-361 cells covered 5.14%, 19.25%, and 23.43% of the scratched area at 24, 48, and 72 h after scratching, respectively (lower panel in Fig. 5A, B). In contrast, 7,8-DHF untreated melanoma cell lines showed area covered with 5%, 30.79%, and 92.03% cells at 0, 24, and 72 h (SK-MEL-2), and with 3%, 34,83%, and 81.29% at 0, 24, and 72 h (G-361) as shown in Fig. 5B. In both melanoma cells treated with 7,8-DHF, the ratio of wound closure was slightly increased, the cell population and migratory capacity were inhibited in a time-dependent manner, compared to the 7,8-DHF untreated ones, respectively.

### 7,8-DHF down-regulated the expression of ganglioside GD3 in melanoma cells

**Fig. 6:**
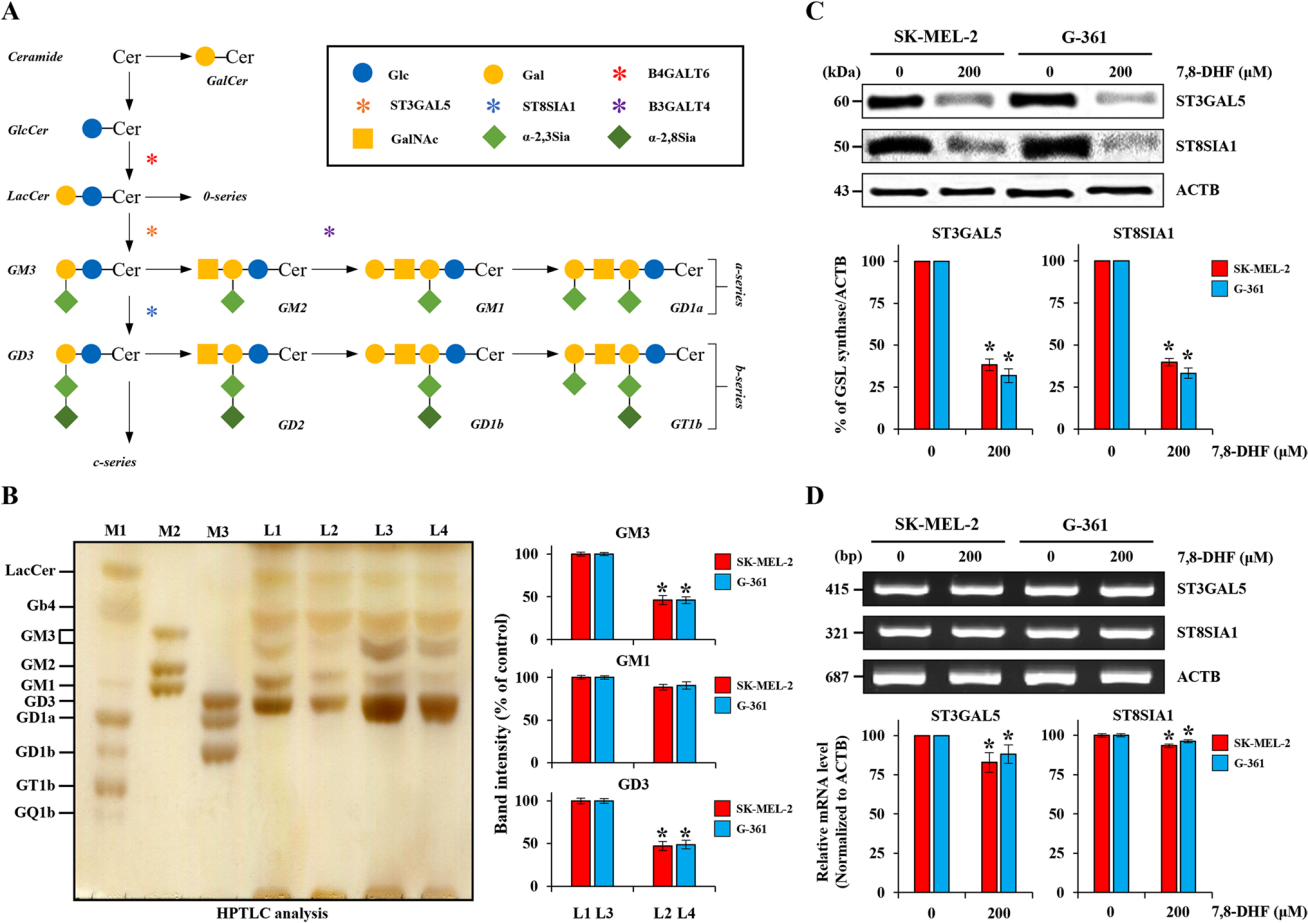
Changes in ganglioside expression in 7,8-DHF treated SK-MEL-2 and G-361 melanoma cells. **A** Common ganglioside biosynthesis. G: ganglioside; M: monosialo; D: disialo; numbers denote carbohydrate sequence. GlcCer: glucosylceramide; LacCer: lactosylceramide; GalNAc: N-acetylgalactosamine; B4GALT6, beta-1,4-galactosyltransferase 6; ST3GAL5, ST3 beta-galactoside alpha-2,3-sialyltransferase 5; B3GALT4, beta-galactosyltransferase 4; ST8SIA1, ST8 alpha-N-acetylneuraminide alpha-2,8-sialyltransferase 1. **B** HPTLC analysis of gangliosides (10 µg/µL) in untreated SK-MEL-2 cells (L1), SK-MEL-2 cells treated with 7,8-DHF (200 µM for 24 h) (L2), untreated G-361 cells (L3), and G-361 cells treated with 7,8-DHF (200 µM for 24 h) (L4). M, marker; L, line; M1 adult rat brain standard gangliosides; M2 and M3, ganglioside standard mixture marker (left panel); the quantification of band intensity for GM3, GM1, and GD3 in SK-MEL-2 and G-361 cells (right panel). Data represent the mean ± SD (*n* = 3, ^*^p < 0.05). **C** Western blot analysis and **D** RT-PCR analysis of the gangliosides GM3 and GD3 synthase was examined, respectively. Red square and blue square indicate SK-MEL-2 and G-361 cells, respectively. ACTB was used as a loading control. ACTB; β-actin. All data were presented as mean percentage levels ± SD (*n* = 3, ^*^p < 0.05)

The ganglioside plays important roles in carcinogenesis. The structures and parts of the ganglioside biosynthesis process as shown in Fig. 6A. Among a variety of gangliosides, GM3 and GD3 are involved in the cell growth, proliferation, cell mobility, migration, invasion, and cell–cell and cell–matrix interaction of many types of cancer cells [32, 48]. To further investigate whether 7,8-DHF causes changes in the ganglioside expression profiles of SK-MEL-2 and G-361 cells, we performed HPTLC analysis. As shown in Fig. 6B, the expression levels of GM3 and GD3 in melanoma SK-MEL-2 and G-361 cells were markedly decreased by 7,8-DHF (200 µM, 24 h) (Fig. 6B). In contrast, in non-tumoral epidermal HaCaT cells, there was no significant difference between the control and 7,8-DHF-treated groups (Additional file 1: Fig. S1A). To further determine the effect of 7,8-DHF on the protein expression of ganglioside synthases, melanoma cells were treated with 7,8-DHF (200 µM for 24 h). As shown in Fig. 6C, 7,8-DHF significantly reduced the protein levels of ST3 beta-galactoside alpha-2,3-sialyltransferase 5 (ST3GAL5; ganglioside GM3 synthase) and ST8 alpha-N-acetyl-neuraminide alpha-2,8-sialyltransferase 1 (ST8SIA1; ganglioside GD3 synthase). However, 7,8-DHF did not suppress ST3GAL5 and ST8SIA1 mRNA expression in SK-MEL-2 or G-361 melanoma cells (Fig. 6D). In HaCaT cells, there were no significant differences in the protein and mRNA levels of ganglioside synthases between the control and 7,8-DHF-treated groups (Fig. 2A and Additional file 1: Fig. S2B).

## Discussion

Malignant melanoma is an increasingly prevalent and fatal form of skin cancer. Despite being rare in the past, its incidence and mortality rates have risen significantly in recent years [3, 4, 36]. To avoid the harmful side effects and unexpected reactions associated with synthetic chemical, it is crucial to investigate therapeutic approaches for melanoma. Therefore, the discovery of effective anti-melanoma agents and specific biomarkers are of great importance (Additional file 2).

Basically, 7,8-dihydroxyflavone (7,8-DHF) was suggested in many studies to provide anti-oxidant effects and anti-carcinogenesis including the development and progression in different cancer types [14, 37, 38]. In the present study, we confirmed that 7,8-DHF effectively induces dose- and time-dependent melanoma cell death, consistent with previous studies on the anti-melanoma properties of 7,8-DHF [11, 12, 20, 21]. However, the absolute treatment concentration setting varied due to the intra-tumor heterogeneity within melanoma caused by genetic mutations acquired irregularly [39–44].

Anti-cancer drugs are known to induce various mechanisms, such as the checkpoint in cell-cycles, in cancer cells. Specifically, we confirmed that treatment with 7,8-DHF caused G2/M phase cell-cycle arrests in both melanoma cells, which is similar to previous studies [19, 45, 46]. Furthermore, mitochondrial damage is followed by a decrease in mitochondrial membrane potential and the expression levels of the proteins [47]. Changes in mitochondrial function during cancer progression play an important role in the regulation and initiation of apoptosis [48]. In the present study, the decrease in mitochondrial membrane potential and changes in the expression of mitochondrial apoptosis pathway-related proteins clearly showed the anti-melanoma ability of 7,8-DHF in the present study. In squamous carcinoma cells, it has been reported that the treatment with methyl-honokiol induces mitochondrial dysfunction in the process of cancer cell death well [49]. Moreover, cancer migration and mobility are highly important for cancer progression and metastasis [50]. We confirmed 7,8-DHF effectively reduced the recovery ability and mobility in both melanoma cells line, which is also similar to previous studies [19, 45].

Ganglioside is closely involved cellular metabolisms including growth, proliferation, differentiation, metastasis, and the general cell apoptosis [22–25]. Normally, ganglioside GD3 and GM3 are highly expressed in skin cancer, ganglioside GM3 in squamous carcinoma, ganglioside GM2 in pancreatic cancer, ganglioside GM3 in colorectal cancer, and ganglioside GD3 in breast cancer [54]. Especially, ganglioside GD3 is reported to be a promising target for immunotherapy in melanoma cancers [22, 23, 51–54]. In addition, ganglioside GD3 activates growth factor receptors that are combined with adhesion signals, resulting in the activation of downstream signaling molecules and in increased growth and invasion of melanoma cells [53, 55] In the present study, we confirmed that the ganglioside GD3 and its synthase were highly decreased in 7,8-DHF-treated melanoma cells, compared to 7,8-DHF-untreated ones. Meanwhile, regulation of the expression of ganglioside GD3 was blocked through mitochondrial changes, decreased activation of caspase 9, and plays a crucial role in the orchestration of apoptosis signals [56], which is also similar to our findings of the present study.

Taken together, the treatment of 7,8-DHF against melanoma cells might have effectual and superior anti-cancer efficacy, indicating its potential as an anti-melanoma therapeutic agent such as a biomarker. Furthermore, ganglioside might be a double-edged sword against melanoma cells; suggesting that 7,8-DHF could be a candidate targeted anti-cancer therapy.

## Supplementary Information

**Additional file 1: Table S1.** Primer sequences used to generate templates for RT-PCR. **Figure S1.** Changes in ganglioside expression in 7,8-DHF-treated SK-MEL-2, G-361, and non-tumoral HaCaT cells. HPTLC analysis of gangliosides in untreated SK-MEL-2 cells (L1), SK-MEL-2 cells treated with 7,8-DHF [200 µM for 24 hours (hr)] (L2), untreated G-361 cells (L3), G-361 cells treated with 7,8-DHF (200 µM for 24 hr) (L4), untreated HaCaT cells (L5), and HaCaT cells treated with 7,8-DHF (200 µM for 24 hr) (L6). M, marker; L, line; M1 adult rat brain standard gangliosides; M2 and M3, ganglioside standard mixture marker (upper panel); the quantification of band intensity for GM3, GM1, and GD3 in SK-MEL-2, G-361, and HaCaT cells (lower panel). Red square, blue square, and black square indicate SK-MEL-2, G-361, and HaCaT cells, respectively. All data presented the mean ± standard deviation (SD) (*n *= 3, ^*^p < 0.05). **Figure S2. **7,8-DHF shows no significant difference on the expression of ganglioside synthases in non-tumoral HaCaT cells **A** Western blot analysis and **B** reverse transcription-polymerase chain reaction analysis of GM3 and GD3 synthase expression in non-tumoral HaCaT cells. ACTB was used as a loading control. ACTB; β-actin. Data were the results of three independent experiments performed in triplicates (*n *= 3, ^*^p < 0.05).

**Additional file 2: Figure S3.** Uncropped images of all blots, gels, and HPTLC. Uncropped images of Figure 3C (A), Figure 3D (B), Figure 4B (C), Figure 6B (D), Figure 6C (E), Figure 6D (F), Supplementary Figure 1 (G), Supplementary Figure 2A (H), Supplementary Figure 2B (I) were shown.

## Data Availability

The datasets used and/or analyzed during the current study are available from the corresponding author upon reasonable request.
