# Correction: 7,8-Dihydroxyflavone induces mitochondrial apoptosis and down-regulates the expression of ganglioside GD3 in malignant melanoma cells

**DOI:** 10.1007/s12672-023-00653-y

**Published:** 2023-04-24

**Authors:** Won Seok Ju, Sang Young Seo, Seong-eun Mun, Kyongtae Kim, Jin Ok Yu, Jae-Sung Ryu, Ji-Su Kim, Young-Kug Choo

**Affiliations:** 1 Department of Biological Science, College of Natural Sciences, Wonkwang University, 460, Iksan-Daero, Iksan-Si, Jeollabuk-Do 54538 Republic of Korea; 2 Animal Biotechnology Division, Rural Development Administration, National Institute of Animal Science, 1500 Kongjwipatjwi-Ro, Iseo-Myeon, Wanju-Gun, Jeonbuk 55365 Republic of Korea; 3 Stem Cell Convergence Research Center, Korea Research Institute of Bioscience and Biotechnology (KRIBB), 125, Gwahak-Ro, Yuseong-Gu, Daejeon, 34141 Republic of Korea; 4 Primate Resources Center (PRC), Korea Research Institute of Bioscience and Biotechnology (KRIBB), 181, Ipsin-Gil, Jeongeup-Si, Jeollabuk-Do 56216 Republic of Korea; 5 Institute for Glycoscience, Wonkwang University, 460, Iksan-Daero, Iksan-Si, Jeollabuk-Do 54538 Republic of Korea

**Correction: Discover Oncology (2023) 14:36** **https://doi.org/10.1007/s12672-023-00643-0**

The original publication of this article was missing the following contribution statement:

- Won Seok Ju and Sang Young Seo contributed equally to this work.

The original article [1] has been updated.
