# Supplementary material for: 7,8-Dihydroxyflavone induces mitochondrial apoptosis and down-regulates the expression of ganglioside GD3 in malignant melanoma cells
Source: Discov Oncol. 2023 Mar 30;14:36. doi: 10.1007/s12672-023-00643-0 (PMC10060447; doi:10.1007/s12672-023-00643-0)
Supplement: Supplementary file 1 — Additional file 1: Table S1. Primer sequences used to generate templates for RT-PCR. Figure S1. Changes in ganglioside expression in 7,8-DHF-treated SK-MEL-2, G-361, and non-tumoral HaCaT cells. HPTLC analysis of gangliosides in untreated SK-MEL-2 cells (L1), SK-MEL-2 cells treated with 7,8-DHF [200 µM for 24 hours (hr)] (L2), untreated G-361 cells (L3), G-361 cells treated with 7,8-DHF (200 µM for 24 hr) (L4), untreated HaCaT cells (L5), and HaCaT cells treated with 7,8-DHF (200 µM for 24 hr) (L6). M, marker; L, line; M1 adult rat brain standard gangliosides; M2 and M3, ganglioside standard mixture marker (upper panel); the quantification of band intensity for GM3, GM1, and GD3 in SK-MEL-2, G-361, and HaCaT cells (lower panel). Red square, blue square, and black square indicate SK-MEL-2, G-361, and HaCaT cells, respectively. All data presented the mean ± standard deviation (SD) (n = 3, *p < 0.05). Figure S2. 7,8-DHF shows no significant difference on the expression of ganglioside synthases in non-tumoral HaCaT cells A Western blot analysis and B reverse transcription-polymerase chain reaction analysis of GM3 and GD3 synthase expression in non-tumoral HaCaT cells. ACTB was used as a loading control. ACTB; β-actin. Data were the results of three independent experiments performed in triplicates (n = 3, *p < 0.05). [file 12672_2023_643_MOESM1_ESM.docx]

**7,8-Dihydroxyflavone induces mitochondrial apoptosis and down-regulates the expression of ganglioside GD3 in malignant melanoma cells**

Won Seok Ju^1,2,†^, Sang Young Seo^1,†^, Seong-eun Mun^1^, Kyongtae Kim^1^, Jin Ok Yu^1^, Jae-Sung Ryu^3^, Ji-Su Kim^4^, and Young-Kug Choo^1,5,*^

^1^Department of Biological Science, College of Natural Sciences, Wonkwang University, 460, Iksan-daero, Iksan-si, Jeollabuk-do 54538, Republic of Korea

^2^Animal Biotechnology Division, National Institute of Animal Science, Rural Development Administration, 1500 Kongjwipatjwi-ro, Iseo-myeon, Wanju-gun, Jeonbuk 55365, Republic of Korea

^3^Stem Cell Convergence Research Center, Korea Research Institute of Bioscience and Biotechnology (KRIBB), 125, Gwahak-ro, Yuseong-gu, Daejeon, 34141, Republic of Korea

^4^Primate Resources Center (PRC), Korea Research Institute of Bioscience and Biotechnology (KRIBB), 181, Ipsin-gil, Jeongeup-si, Jeollabuk-do 56216, Republic of Korea

^5^Institute for Glycoscience, Wonkwang University, 460, Iksan-daero, Iksan-si, Jeollabuk-do 54538, Republic of Korea

^†^These authors contributed equally to this work.

^*^Correspondence to: Young-Kug Choo, Department of Biological Science, College of Natural Sciences, Wonkwang University, 460, Iksan-daero, Iksan-si, Jeollabuk-do 54538, Republic of Korea, Tel.: +82-63-850-6087, Fax: +82-63-857-8837, E-mail: ykchoo@wku.ac.kr

**Fig. S1**


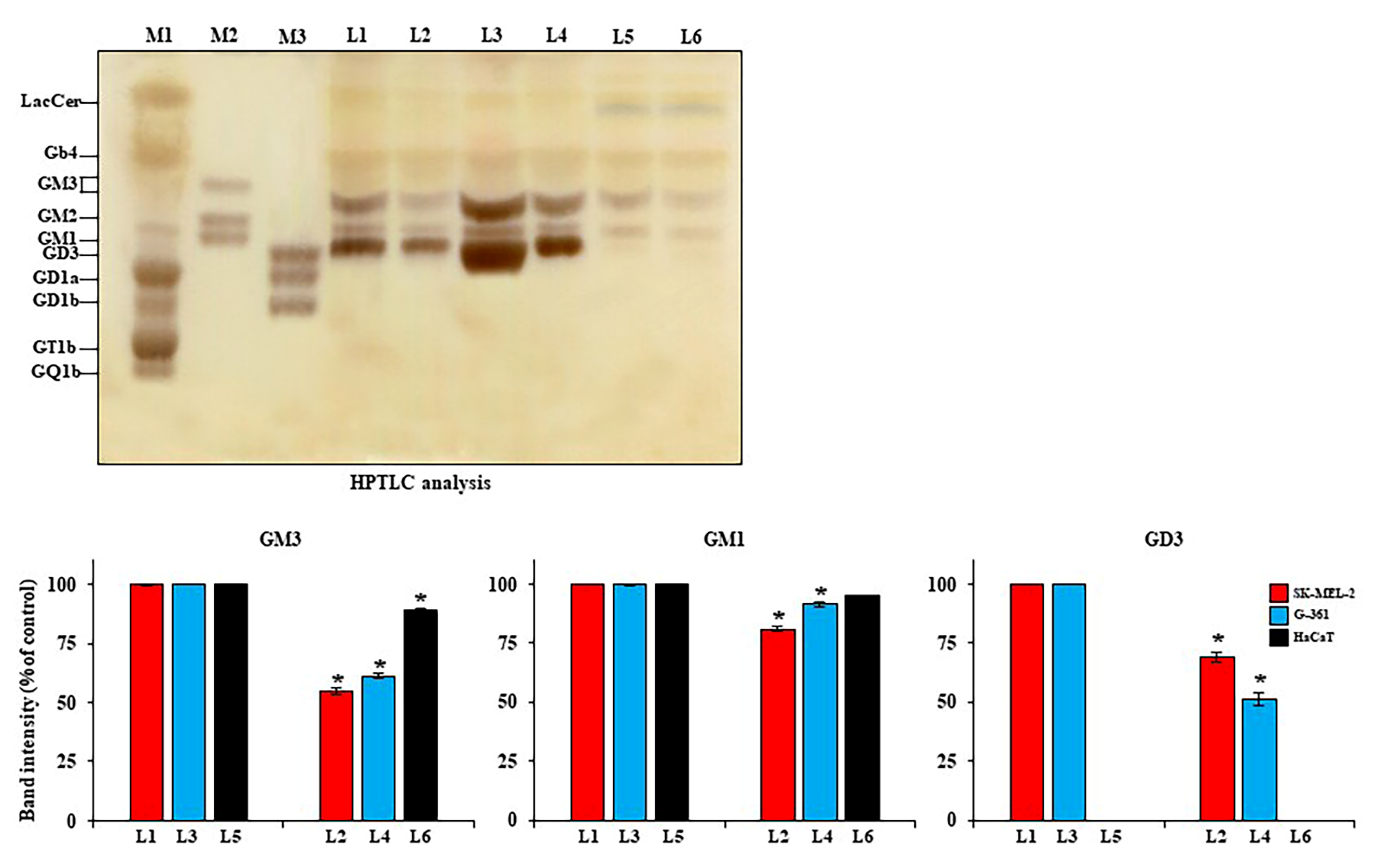


**Fig. S2**


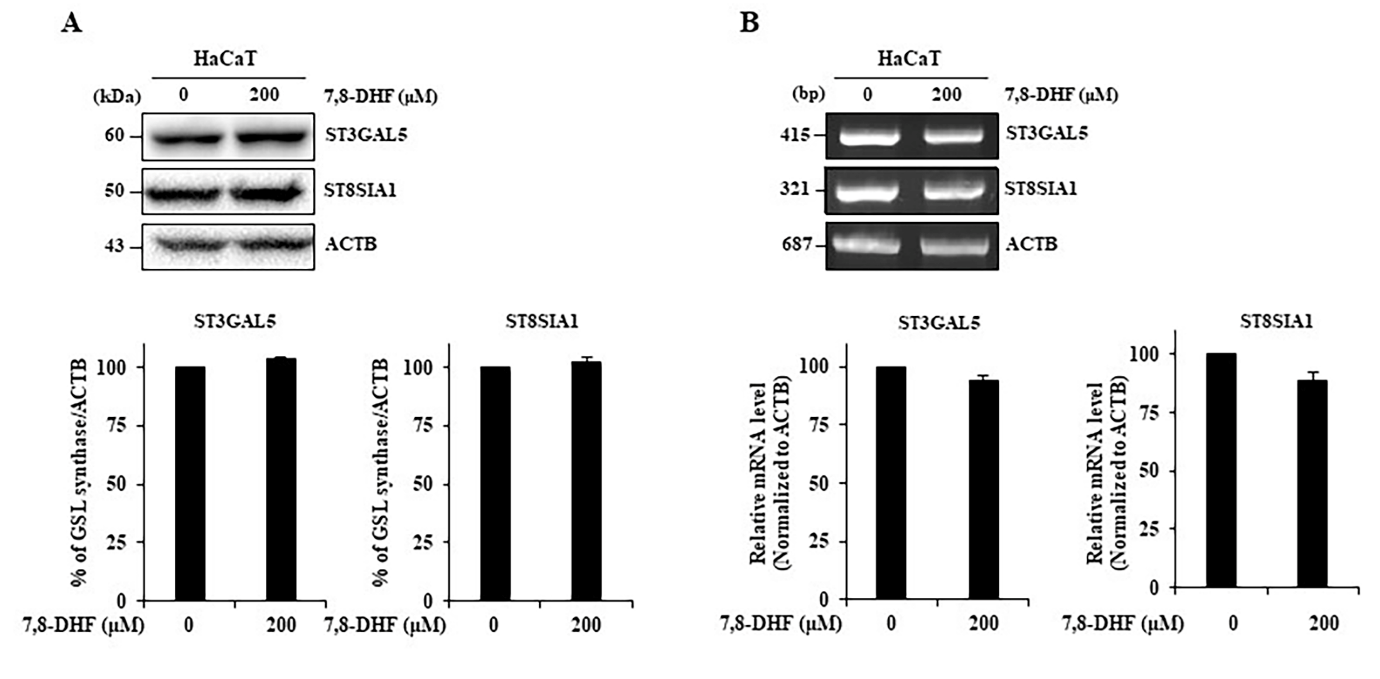


**Supplementary Figure legends**

**Fig. S1** Changes in ganglioside expression in 7,8-DHF-treated SK-MEL-2, G-361, and non-tumoral HaCaT cells. HPTLC analysis of gangliosides in untreated SK-MEL-2 cells (L1), SK-MEL-2 cells treated with 7,8-DHF [200 µM for 24 hours (hr)] (L2), untreated G-361 cells (L3), G-361 cells treated with 7,8-DHF (200 µM for 24 hr) (L4), untreated HaCaT cells (L5), and HaCaT cells treated with 7,8-DHF (200 µM for 24 hr) (L6). M, marker; L, line; M1 adult rat brain standard gangliosides; M2 and M3, ganglioside standard mixture marker (upper panel); the quantification of band intensity for GM3, GM1, and GD3 in SK-MEL-2, G-361, and HaCaT cells (lower panel). Red square, blue square, and black square indicate SK-MEL-2, G-361, and HaCaT cells, respectively. All data presented the mean ± standard deviation (SD) (*n* = 3, ^*^*p* < 0.05).

**Fig. S2** 7,8-DHF shows no significant difference on the expression of ganglioside synthases in non-tumoral HaCaT cells **A** Western blot analysis and **B** reverse transcription-polymerase chain reaction analysis of GM3 and GD3 synthase expression in non-tumoral HaCaT cells. ACTB was used as a loading control. ACTB; β-actin. Data were the results of three independent experiments performed in triplicates (*n* = 3, ^*^*p* < 0.05)

**Table S1**

Primer sequences used to generate templates for RT-PCR.

| **Gene** | **Description** | **Species** | **Primer (5′→3′)** | **Reaction condition** | **Size (bp)** |
| --- | --- | --- | --- | --- | --- |
| ST3GAL5 | ST3 beta-galactoside alpha-2,3-sialyltransferase 5 | *Homo sapiens* | F: CCC TGA ACC AGT TCG ATG TT  R: GTG GCT AAG ACA ACG GCA AT | Denaturation: 30 s at 95 °C  Annealing: 30 s at 62 °C  Extension: 30 s at 72 °C  Reaction cycles: 35 | 415 |
| ST8SIA1 | ST8 alpha-N-acetylneuraminide alpha-2,8-sialyltransferase 1 | *Homo sapiens* | F: GCA ATC TCC CTC CTC CTT TGT CA  R: GCT TGG CAT GGA TTC CTC TA |  | 321 |
| ACTB | β-Actin | *Homo sapiens* | F: CGC AAG TAC TCC GTG TGG AT  R: GTC AGT GTA CAG GTA AGC CCT G |  | 687 |
