# Supplementary material for: 7,8-Dihydroxyflavone induces mitochondrial apoptosis and down-regulates the expression of ganglioside GD3 in malignant melanoma cells
Source: Discov Oncol. 2023 Mar 30;14:36. doi: 10.1007/s12672-023-00643-0 (PMC10060447; doi:10.1007/s12672-023-00643-0)
Supplement: Supplementary file 2 — Additional file 2: Figure S3. Uncropped images of all blots, gels, and HPTLC. Uncropped images of Figure 3C (A), Figure 3D (B), Figure 4B (C), Figure 6B (D), Figure 6C (E), Figure 6D (F), Supplementary Figure 1 (G), Supplementary Figure 2A (H), Supplementary Figure 2B (I) were shown. [file 12672_2023_643_MOESM2_ESM.docx]

**7,8-Dihydroxyflavone induces mitochondrial apoptosis and down-regulates the expression of ganglioside GD3 in malignant melanoma cells**

Won Seok Ju^1,†^, Sang Young Seo^1,†^, Seong-eun Mun^1^, Jin Ok Yu^1^, Jae-Sung Ryu^2^, Ji-Su Kim^3^, and Young-Kug Choo^1,4,*^

^1^Department of Biological Science, College of Natural Sciences, Wonkwang University, 460, Iksan-daero, Iksan-si, Jeollabuk-do 54538, Republic of Korea

^2^Stem Cell Convergence Research Center, Korea Research Institute of Bioscience and Biotechnology (KRIBB), 125, Gwahak-ro, Yuseong-gu, Daejeon, 34141, Republic of Korea

^3^Primate Resources Center (PRC), Korea Research Institute of Bioscience and Biotechnology (KRIBB), 181, Ipsin-gil, Jeongeup-si, Jeollabuk-do 56216, Republic of Korea

^4^Institute for Glycoscience, Wonkwang University, 460, Iksan-daero, Iksan-si, Jeollabuk-do 54538, Republic of Korea

^*^Correspondence to: Young-Kug Choo, Department of Biological Science, College of Natural Sciences, Wonkwang University, 460, Iksan-daero, Iksan-si, Jeollabuk-do 54538, Republic of Korea, Tel.: +82-63-850-6087, Fax: +82-63-857-8837, E-mail: ykchoo@wku.ac.kr

**Additional file Figure 3**

| **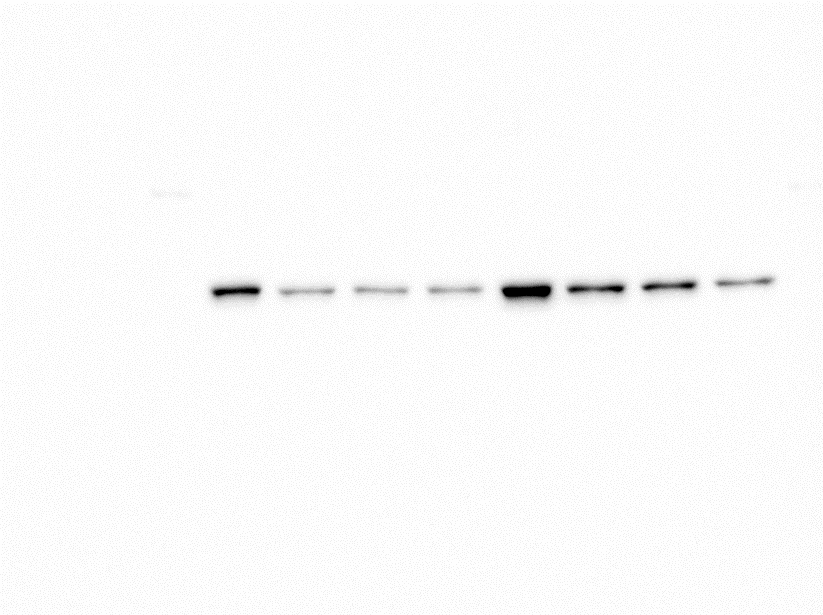**  A  **33 kDa** |
| --- |
| **CDK1** |
| **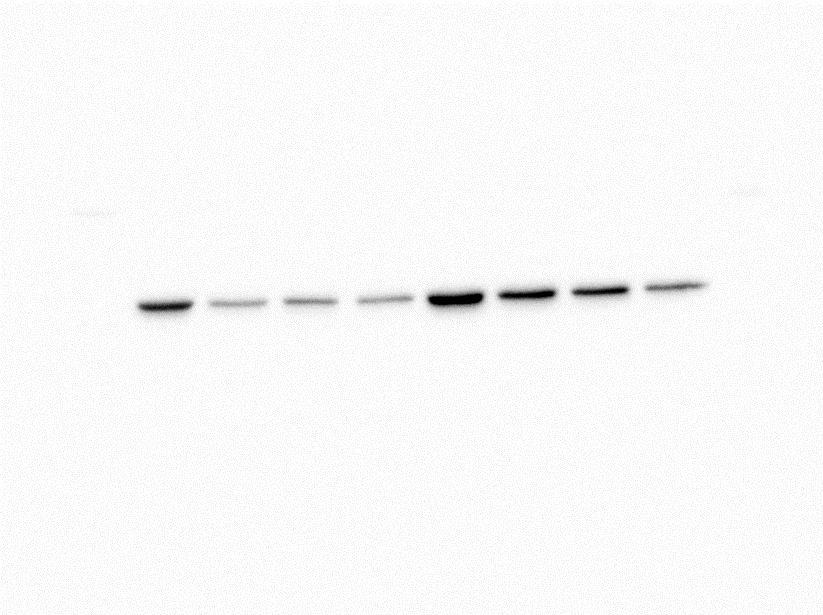**  **60 kDa** |
| **Cyclin B** |
| **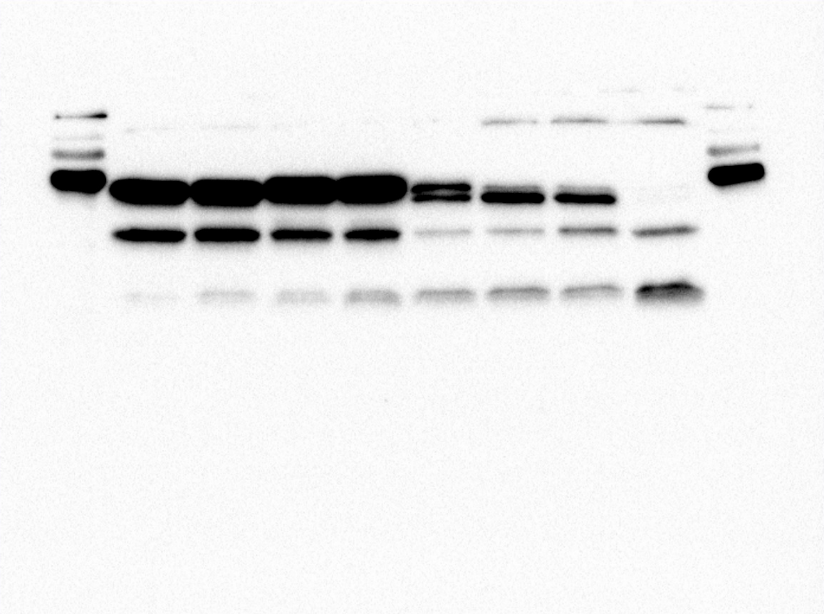**  **40 kDa**  **50 kDa**  **35 kDa**  **25 kDa**  **21 kDa** |
| **p21** |
| **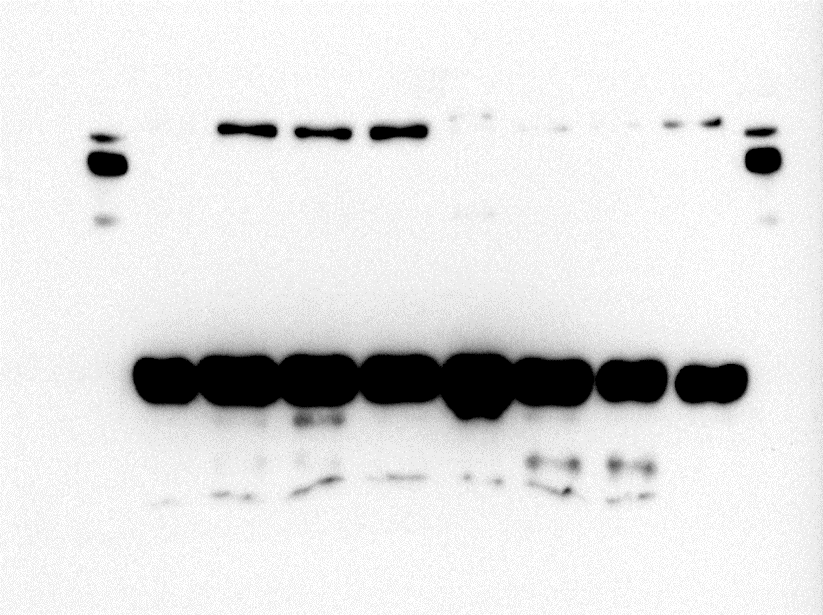**  **25 kDa**  **40 kDa**  **50 kDa**  **53 kDa** |
| **p53** |
| **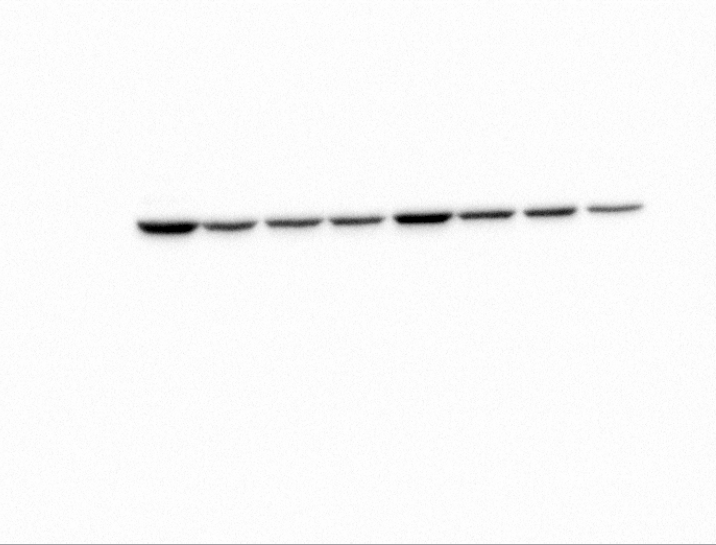**  **40 kDa** |
| **Mcl-1** |
| **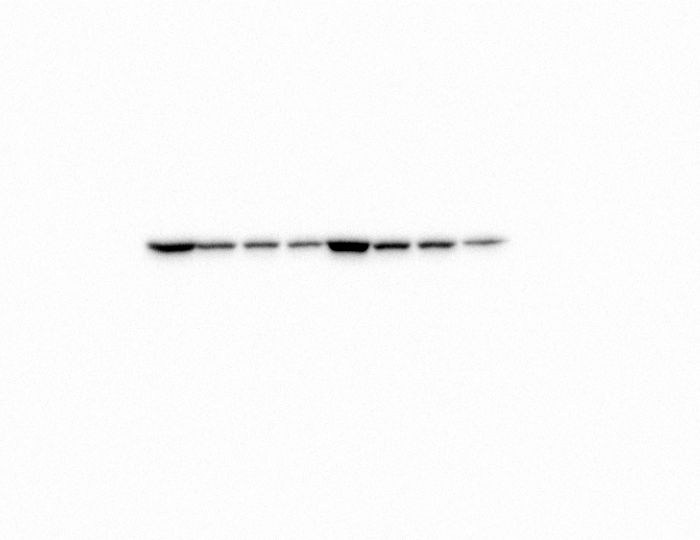**  **17 kDa** |
| **Survivin** |
| **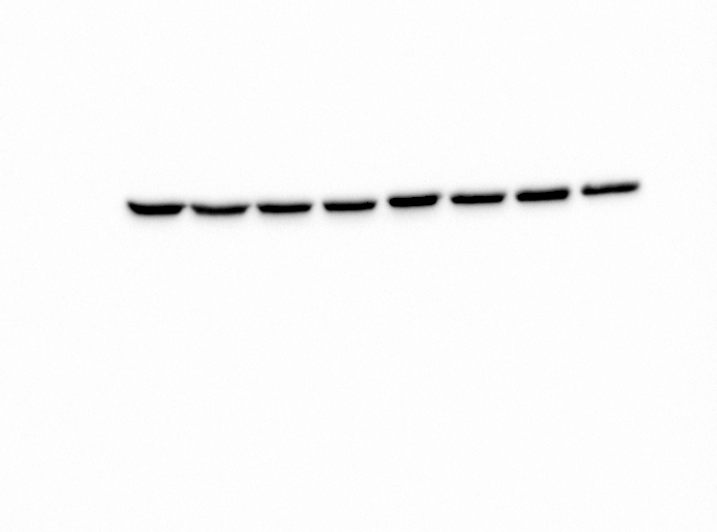**  **43 kDa** |
| **ACTB** |

B

| **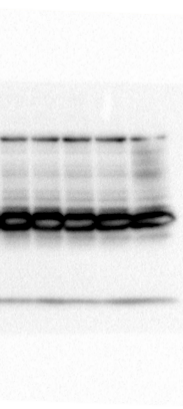**  **23 kDa** | **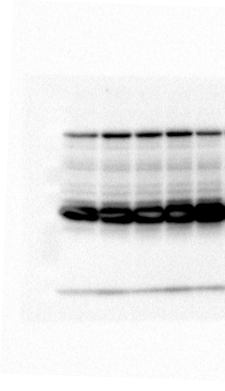**  **23 kDa** |
| --- | --- |
| **Bax (SK-MEL-2)** | **Bax (G-361)** |
| **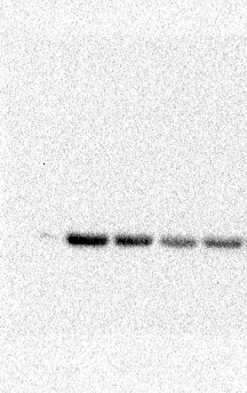**  **30 kDa** | **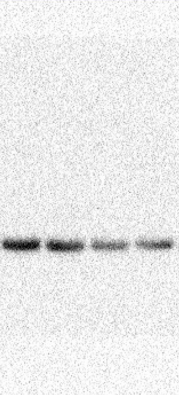**  **30 kDa** |
| **Bcl-xL (SK-MEL-2)** | **Bcl-xL (G-361)** |
| **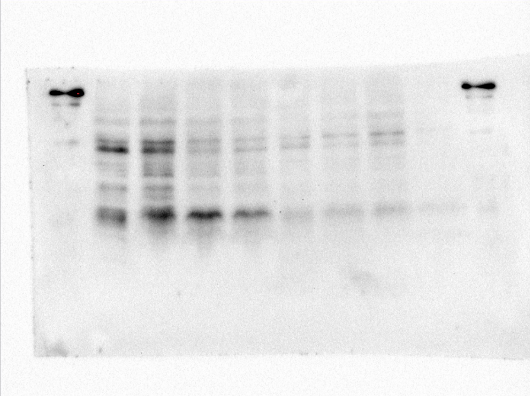**  **55 kDa**  **70 kDa**  **32 kDa** | |
| **Caspase-3** | |
| **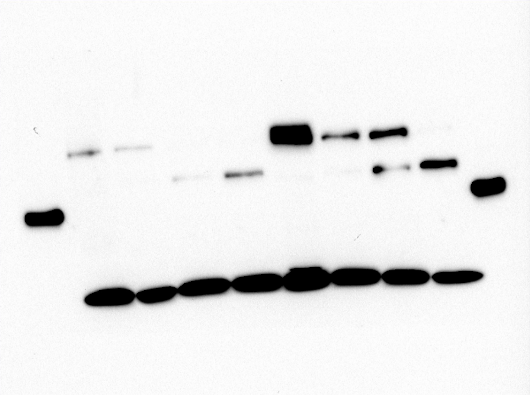**  **70 kDa**  **89 kDa**  **116 kDa** | |
| **PARP/c-PARP** | |
| **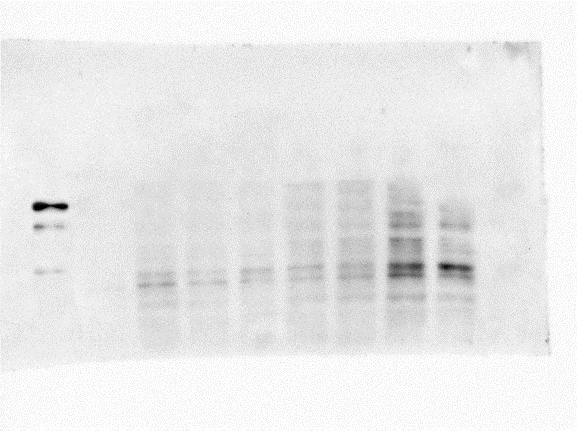**  **35 kDa**  **55 kDa**  **32 kDa** | |
| **Cleaved caspase-3** | |
| **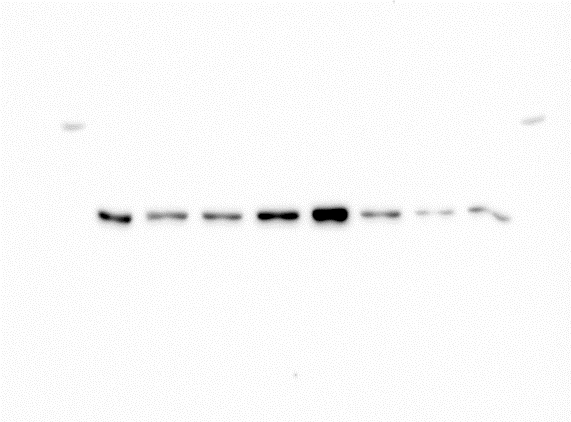**  **55 kDa**  **22 kDa** | |
| **Bid** | |
| **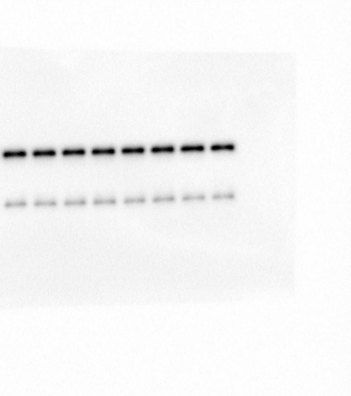**  **43 kDa** | |
| **ACTB** | |

| **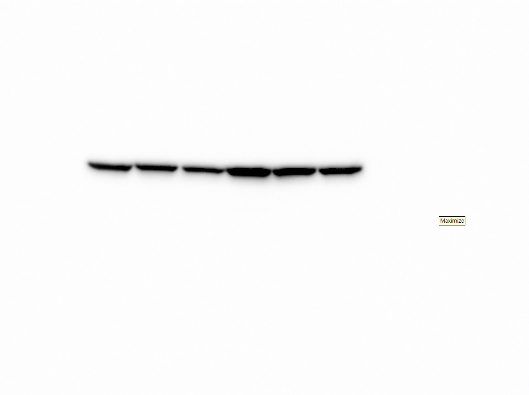**  C  **43 kDa** | **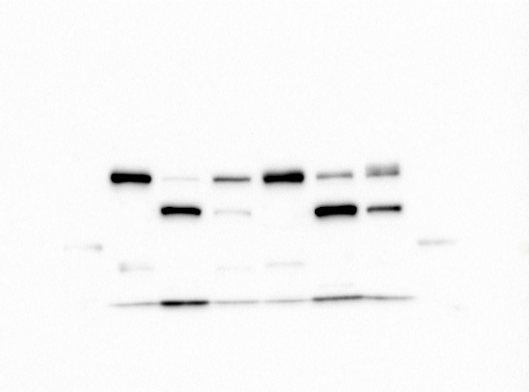**  **70 kDa**  **116 kDa**  **89 kDa** |
| --- | --- |
| **ACTB** | **PARP/c-PARP** |
| **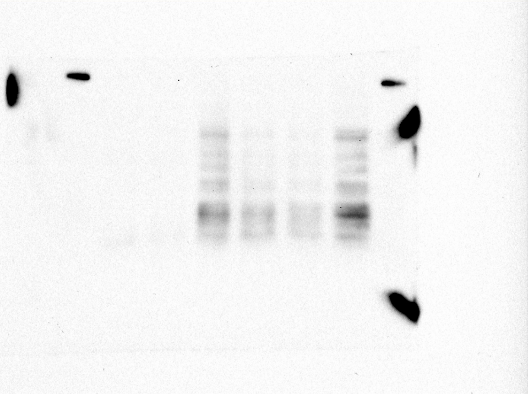**  **55 kDa**  **70 kDa**  **32 kDa** | **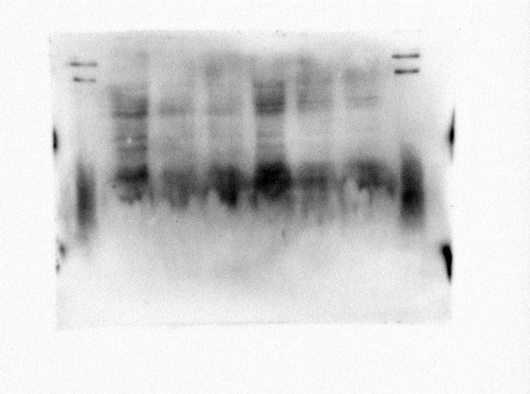**  **55 kDa**  **70 kDa**  **32 kDa** |
| **Cleaved caspase-3** | **Caspase-3** |
| **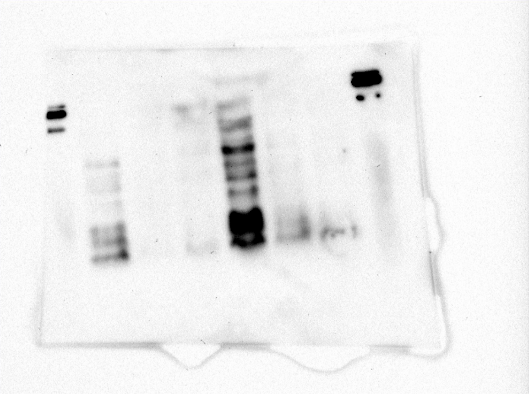**  **60 kDa**  **70 kDa**  **46 kDa** | **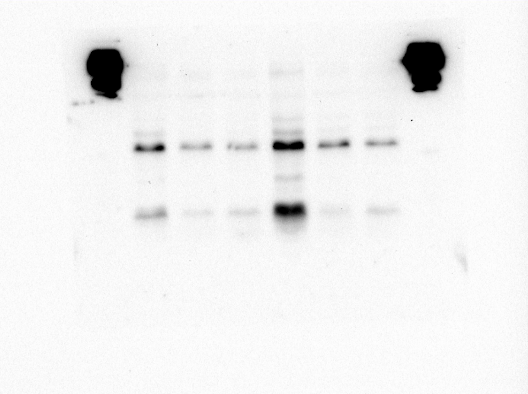**  **55 kDa**  **70 kDa**  **30 kDa** |
| **Caspase-9** | **Bcl-xL** |
| **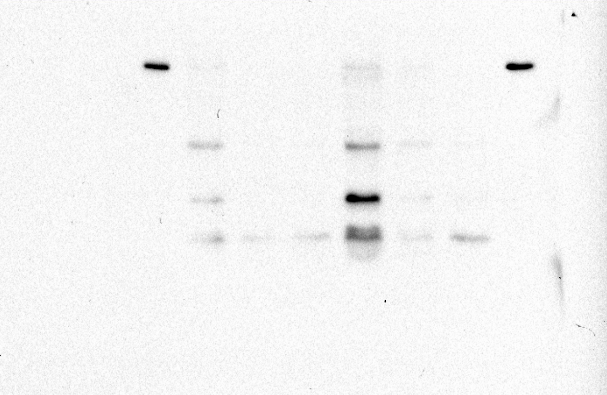**  **70 kDa**  **22 kDa** | **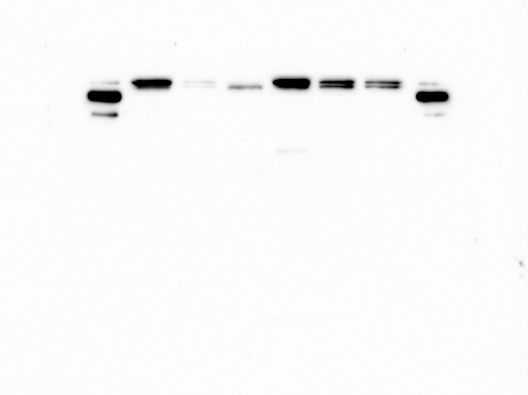**  **15 kDa**  **20 kDa**  **23 kDa** |
| **Bid** | **Bax (Mito.)** |
| **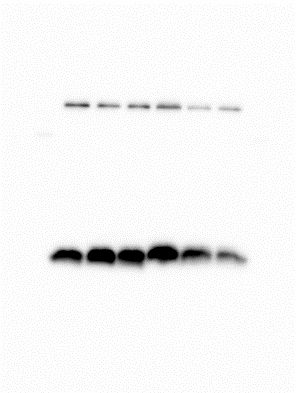**  **15 kDa** | **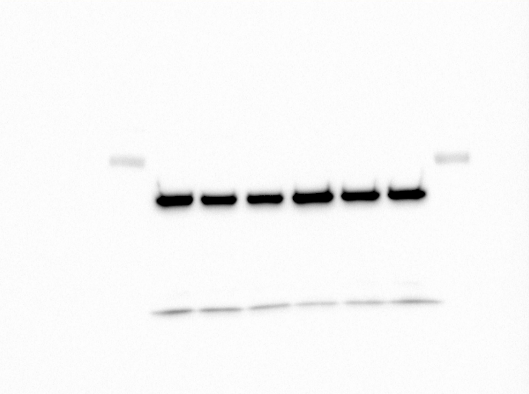**  **70 kDa**  **60 kDa** |
| **Cytochrome C (Mito.)** | **MTCO1 (Mito.)** |
| **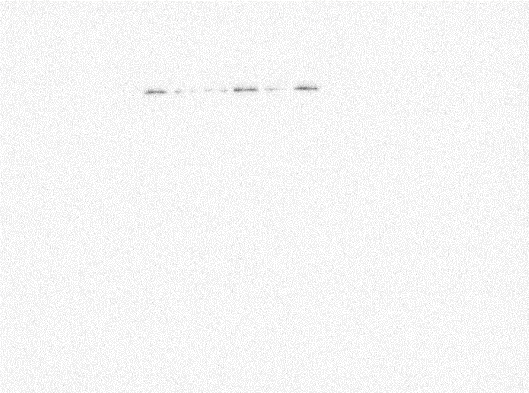**  **23 kDa** | **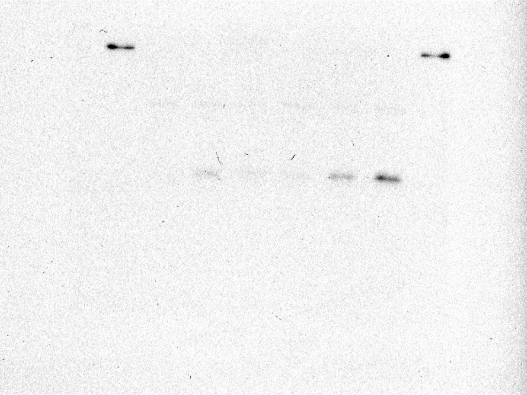**  **25 kDa**  **15 kDa** |
| **Bax (Cyto.)** | **Cytochrome C (Cyto.)** |
| **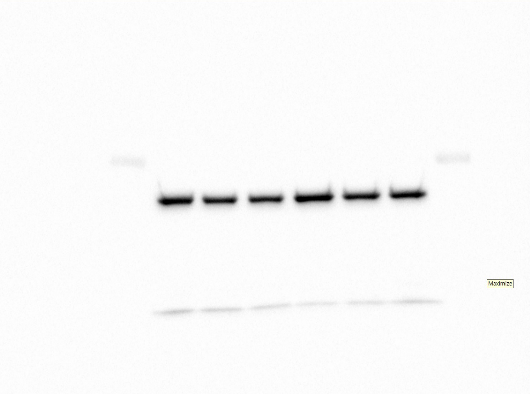**  **70 kDa**  **55 kDa** |  |
| **β-tubulin (Cyto.)** |  |

D


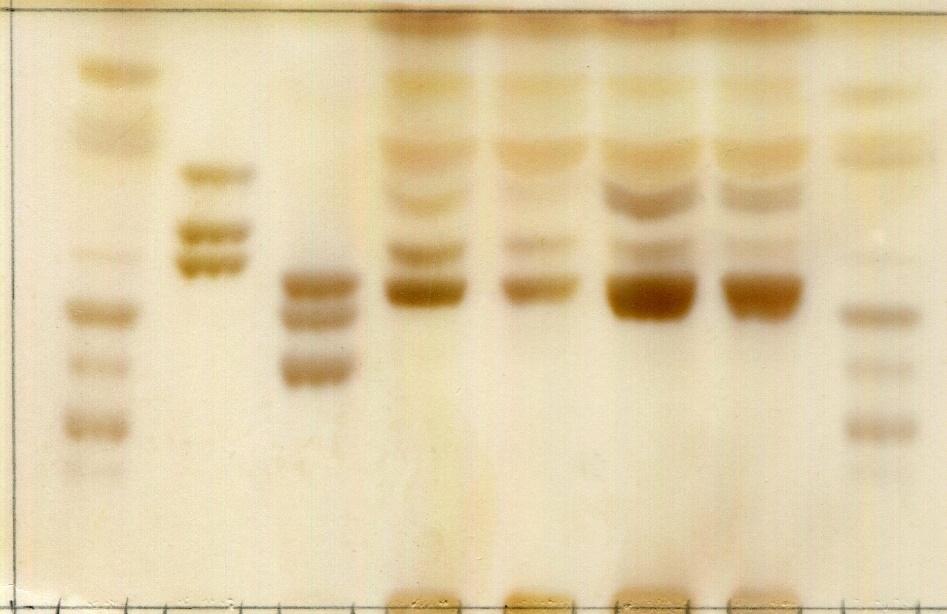


E

| **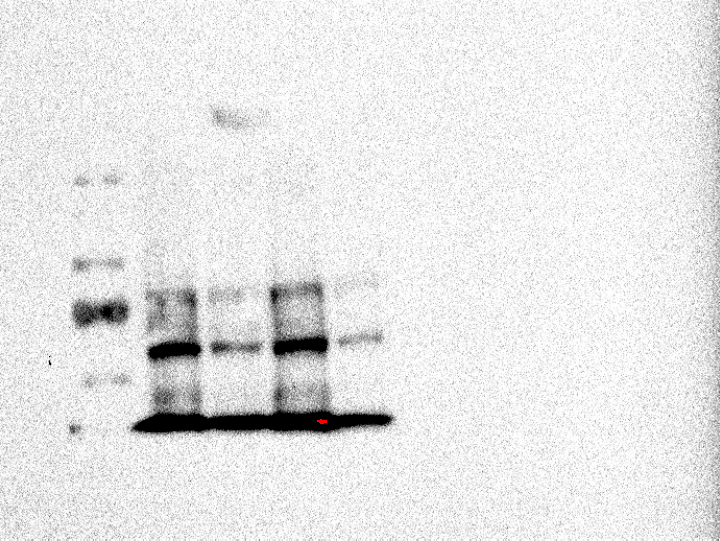**  **72 kDa**  **43 kDa**  **55 kDa**  **50 kDa** |
| --- |
| **ST8SIA1** |
| **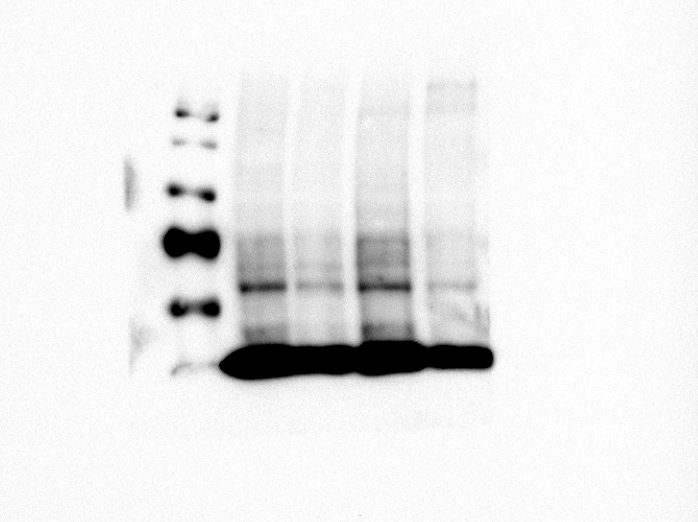**  **95 kDa**  **55 kDa**  **72 kDa**  **60 kDa** |
| **ST3GAL5** |
| **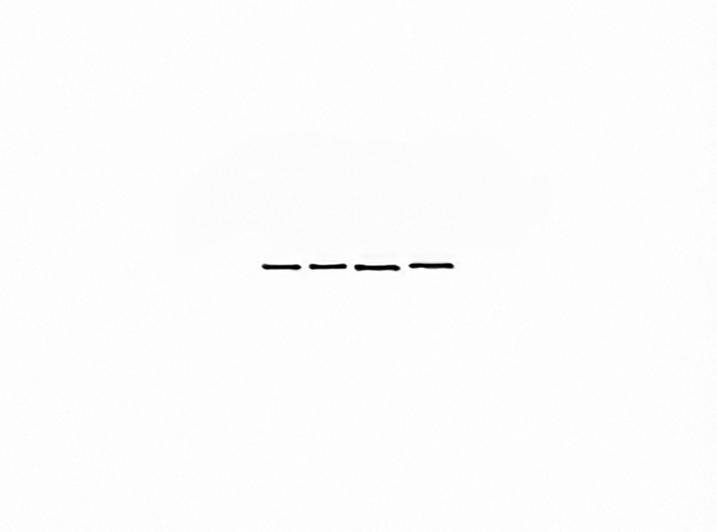**  **43 kDa** |
| **ACTB** |

F

| **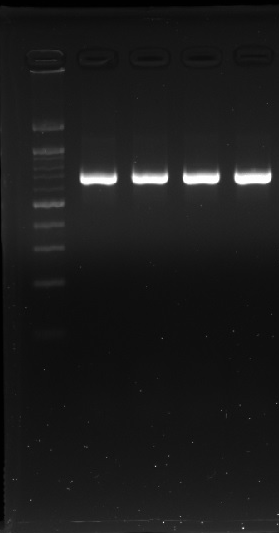**  **242 bp**  **622 bp**  **307 bp**  **404 bp**  **527 bp**  **415 bp** | **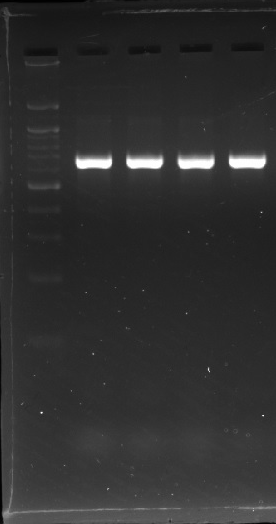**  **527 bp**  **160 bp**  **242 bp**  **404 bp**  **307 bp**  **312 bp** |
| --- | --- |
| **ST3GAL5**  **1000 bp** | **ST8SIA1** |
| **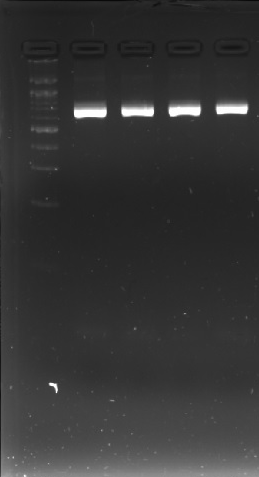**  **900 bp**  **800 bp**  **700 bp**  **400 bp**  **500 bp**  **600 bp**  **687 bp** |  |
| **ACTB** |  |

G


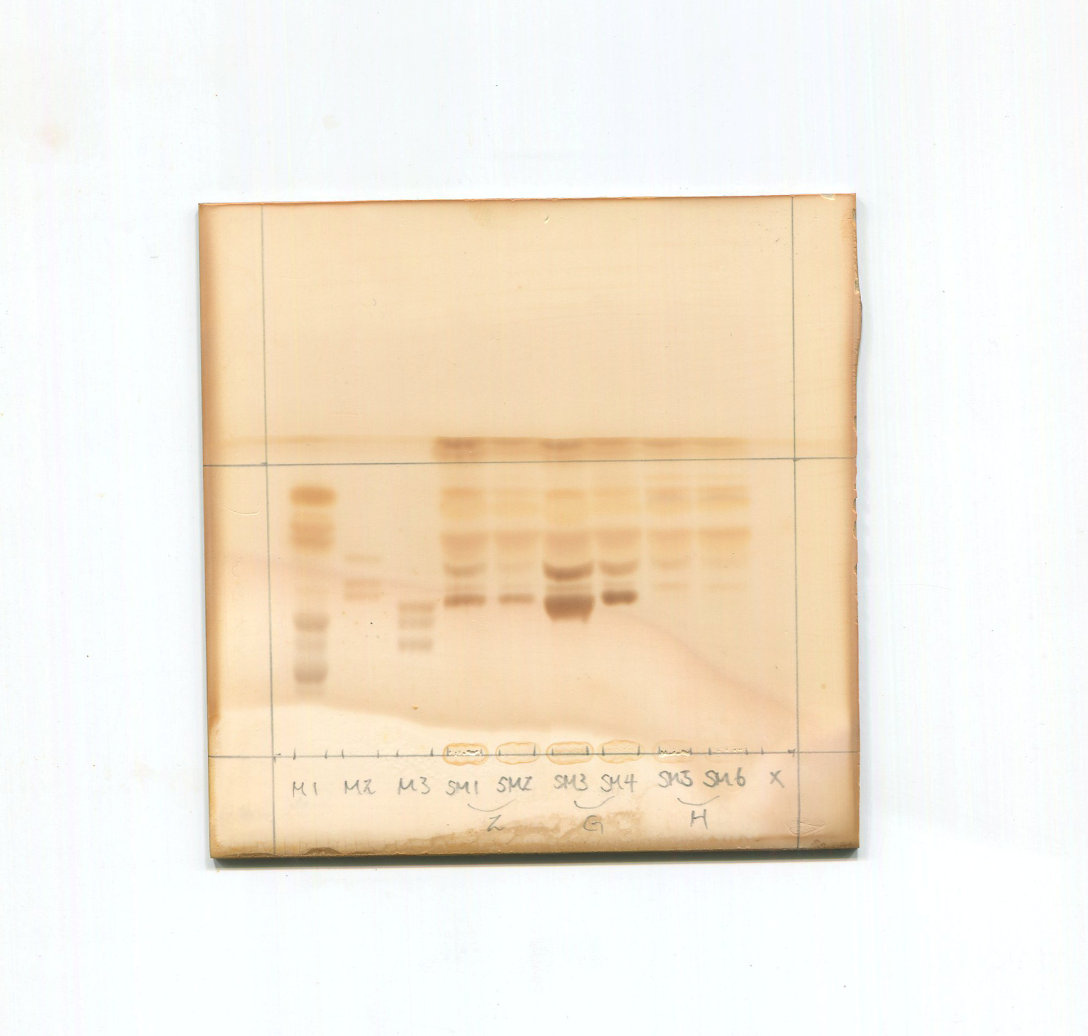


**43 kDa**

H

| **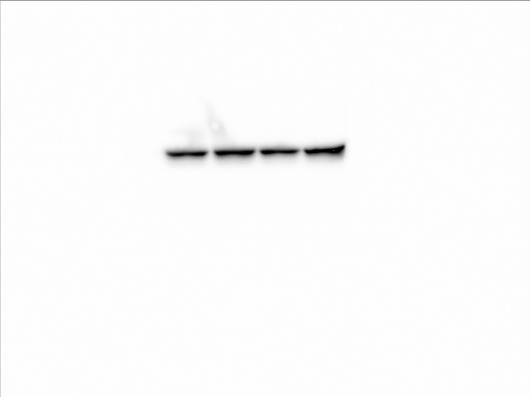**  **50 kDa**  **60 kDa** | **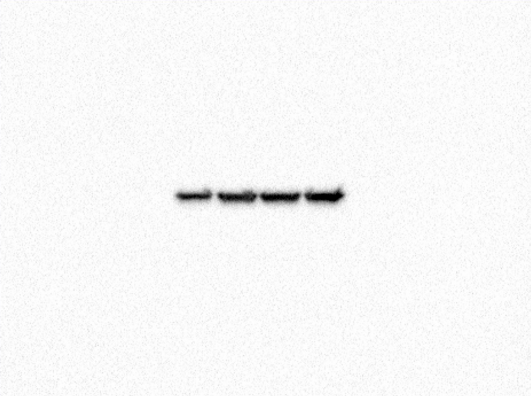** |
| --- | --- |
| **ST3GAL5** | **ST8SIA1** |
| **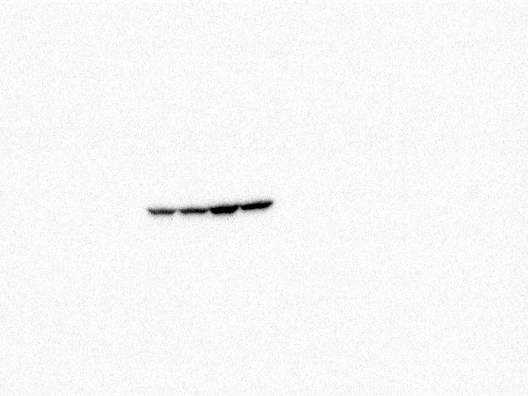** |  |
| **ACTB** |  |

I

| **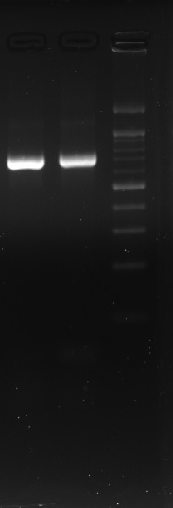**  **1000 bp**  **600 bp**  **500 bp**  **100 bp**  **200 bp**  **300 bp**  **400 bp**  **415 bp** | **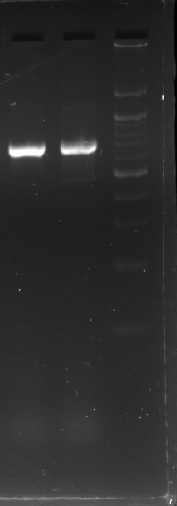**  **160 bp**  **242 bp**  **622 bp**  **527 bp**  **404 bp**  **307 bp**  **312 bp** |
| --- | --- |
| **ST3GAL5** | **ST8SIA1** |
| **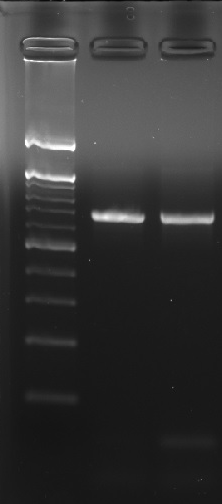**  **1000 bp**  **900 bp**  **800 bp**  **700 bp**  **600 bp**  **300 bp**  **400 bp**  **500 bp**  **687 bp** |  |
| **ACTB** |  |

**Additional file Figure S3.** **Uncropped images of all blots, gels, and HPTLC**. Uncropped images of Figure 3C (A), Figure 3D (B), Figure 4B (C), Figure 6B (D), Figure 6C (E), Figure 6D (F), Supplementary Figure 1 (G), Supplementary Figure 2A (H), Supplementary Figure 2B (I) were shown.
